# Cluster Differentiating 36 (CD36) Deficiency Attenuates Obesity-Associated Oxidative Stress in the Heart

**DOI:** 10.1371/journal.pone.0155611

**Published:** 2016-05-19

**Authors:** Mohamed Gharib, Huan Tao, Thomas V. Fungwe, Tahar Hajri

**Affiliations:** 1 Department of Surgery, Hackensack University Medical Center, New Jersey 07601, United States of America; 2 Division of Cardiovascular Medicine, Vanderbilt University, Nashville, Tennessee 37212, United States of America; 3 Nutritional Sciences, Howard University, Washington DC 20059, United States of America; Maastricht University, NETHERLANDS

## Abstract

**Rationale:**

Obesity is often associated with a state of oxidative stress and increased lipid deposition in the heart. More importantly, obesity increases lipid influx into the heart and induces excessive production of reactive oxygen species (ROS) leading to cell toxicity and metabolic dysfunction. Cluster differentiating 36 (CD36) protein is highly expressed in the heart and regulates lipid utilization but its role in obesity-associated oxidative stress is still not clear.

**Objective:**

The aim of this study was to determine the impact of CD36 deficiency on cardiac steatosis, oxidative stress and lipotoxicity associated with obesity.

**Methods and Results:**

Studies were conducted in control (Lean), obese leptin-deficient (Lep^ob/ob^) and leptin-CD36 double null (Lep^ob/ob^CD36^-/-^) mice. Compared to lean mice, cardiac steatosis, and fatty acid (FA) uptake and oxidation were increased in Lep^ob/ob^ mice, while glucose uptake and oxidation was reduced. Moreover, insulin resistance, oxidative stress markers and NADPH oxidase-dependent ROS production were markedly enhanced. This was associated with the induction of NADPH oxidase expression, and increased membrane-associated p47^phox^, p67^phox^ and protein kinase C. Silencing CD36 in Lep^ob/ob^ mice prevented cardiac steatosis, increased insulin sensitivity and glucose utilization, but reduced FA uptake and oxidation. Moreover, CD36 deficiency reduced NADPH oxidase activity and decreased NADPH oxidase-dependent ROS production. In isolated cardiomyocytes, CD36 deficiency reduced palmitate-induced ROS production and normalized NADPH oxidase activity.

**Conclusions:**

CD36 deficiency prevented obesity-associated cardiac steatosis and insulin resistance, and reduced NADPH oxidase-dependent ROS production. The study demonstrates that CD36 regulates NADPH oxidase activity and mediates FA-induced oxidative stress.

## Introduction

Obesity is often associated with multiple morbidities and a state of oxidative stress, defined as excess production of reactive oxygen species (ROS) relative to antioxidant defense [1]. More importantly, excessive ROS production has been implicated in oxidative damages of lipids and proteins, and initiation of cardiovascular pathological conditions [1], [2]. Previous investigations in human and animal models revealed that oxidative stress induced by obesity is linked to cardiac lipid infiltration [3], [4], and plays an important role in metabolic dysregulations[4], [5], [6].

Increasing evidence has established correlative and causative links between high level of blood free fatty acids (FFAs) and increased risk of cardiac lipotoxicity [7], [8]. The heart’s ability to store lipids is limited and although FAs are the main source of energy, increased FA influx may cause lipotoxicity and oxidative stress [3], [9], [10]. Features of cardiac lipotoxicity have been reported in genetically obese animal models such ob/ob and db/db mice and Zucker rat, and were associated with increased lipid accumulation in myocardium causing insulin resistance [11], [12]. In these models, deposition of fat in the heart is followed by oxidative stress and evidence of apoptosis of cardiomyocytes. Although the precise mechanism(s) of action responsible for the initiation of cardiac abnormalities in obesity remains poorly understood, strong evidence implicates excess lipid accumulation in cell toxicity and dysfunction [2], [3], [10].

Apart from FAs availability, the heart is equipped with multiple regulatory mechanisms that contribute to maintaining a sustained supply of lipids as FFAs [13], [14]. In addition to passive diffusion, a protein-facilitated mechanism has been described as an important route of FA delivery in the heart [13], [14]. The cluster differentiation (CD36) protein is one among other candidates that plays a prominent role in delivering long chain FAs to the heart [13], [15], [16]. In fact, silencing CD36 in mice greatly reduced FA delivery to the cell [16], [17], [18], whereas over-expression of CD36 is associated with increased FA uptake and accumulation of lipids in the heart [13]. In obesity, the availability of FAs is increased while the rate of glucose uptake is reduced; leading the heart to utilize even more FAs for its energy needs [5], [9]. This raises questions about the contribution of CD36 under these pathological conditions. Previously, we have shown that CD36 deficiency reduces lipid accumulation in peripheral organs of lean mice [18], but the question whether CD36 expression alters obesity-associated oxidative stress and lipotoxicity is still unknown. Accordingly, we sought to investigate the impact of CD36 deficiency on cardiac lipid accumulation and oxidative stress in obese leptin-deficient mice.

## Materials and Methods

### Animals and ethics statement

Mice deficient in both leptin and CD36 were generated by breeding CD36 deficient mice (CD36^-/-^) with C57BL/6J-Lep^ob/+^ mice (The Jackson Laboratories, Bar Harbor, ME). Double heterozygotes were then mated to generate leptin and CD36 double null (Lep^ob/ob^ CD36^-/-^) mice. Parallel breeding of male and female heterozygous C57BL/6J-Lep^ob/+^ mice generated homozygotes CD36 positive leptin-deficient (Lep^ob/ob^) mice. Previous investigations including ours have examined the phenotype of CD36 null mice generated on the lean C57BL/6J background 16], [17], [18], [19], [20]. In the present study, we investigated the impact of CD36 deficiency on obesity-associated oxidative stress and lipotoxicity in the heart of genetically obese mice. According, these studies were performed in Lep^ob/ob^ and Lep^ob/ob^ CD36^-/-^ mice, while using Lep^ob/+^ mice control mice (Lean) only as a reference. We refer to previous studies in lean CD36 null mice when needed. Mice were 5–6-month-old and were housed in a facility with a 12-h light cycle and fed ad libitum chow (5001; Purina, St. Louis, MO) diets. All procedures were approved by the Institutional Animal Care and Use Committee of Vanderbilt University and Hackensack University Medical Center University.

### Tissue collection

Mice were fasted overnight and then anesthetized with an intra-peritoneal injection of 100 mg/kg ketamine and 10 mg/kg xylazine prior to blood collection by heart puncture. Cardiovascular system was washed with saline and organs were collected and stored at -80°C for later analysis.

#### Glucose and insulin tolerance tests, and in vivo insulin signaling

Glucose tolerance test (GTT) and insulin tolerance test (ITT) were performed using intraperitoneal injection of glucose solution exactly as described previously [21]. To investigate insulin signaling, hearts were removed 10 minutes after intraperitoneal injection of insulin (Sigma), and were homogenized in ice-cold buffer supplemented with protease inhibitors. Homogenates, collected after centrifugation, were used to perform western blots and determine the level of total and active (phosphorylated) Akt and insulin receptor substrate 1 (IRS1) [21].

#### Assays of cardiac lipids and blood parameters

Heart lipids were extracted by conventional chloroform-methanol procedure [21]. Plasma and heart lipid extracts were used to measure triglycerides (TG), free fatty acids (FAs) and phospholipids (PL) using kits from Thermo Scientific ((Middletown, VA) and Wako Pure Chemical Industries (Richmond, VA) [21]. Insulin and glucose in plasma were measured with the ultrasensitive mice insulin ELISA (R&D Systems, Minneapolis) and glucose colorimetric kit (Thermo scientific). To assess systemic oxidative stress, isoprostane levels were measured in plasma using gas chromatographic/negative ion chemical ionization mass spectrometry (Agilent Technologies, Santa Clara, CA) as described in our previous studies [22].

### Uptake and tissue distribution of fluorodeoxyglucose

Glucose uptake was investigated *in vivo* using fluorodeoxyglucose (^18^F-2-FDG) as reported earlier [18]. Once taken by tissues, FDG is not metabolized and is used to investigate glucose uptake [18], [23]. Fasted mice were injected in the lateral tail vein with 200 μl of saline containing 5 μCi fluorodeoxyglucose (^18^F-2-FDG). Blood samples and tissues were collected under anesthesia, and radioactivity was measured in a gamma counter [18]. To adjust for the difference in blood glucose between mice, initial specific activity of blood FDG, calculated at 2 minutes after injection, was used as the 100% value to correct for tissue uptake [18]. Rates of uptake were calculated as percent of injected dose and per gram wet tissue [18].

### Uptake and incorporation of fatty acid analogue ^125^I-BMIPP

The uptake of FA was examined using FA analogue β-methyl-p-123I- Iodophenyl-Pentadecanoic Acid ^125^I-BMIPP as previously published [24]. Briefly, each mice was injected in the lateral tail vein with 200 μl of the radioisotope solution of [^125^I]-BMIPP (15 μCi). Collection of blood and tissues, measurements of radioactivity and calculation of uptake were performed as previously published [24]. To analyze FA incorporation in newly formed lipids, lipid were extracted from tissue aliquots and were used to separate lipid classes with thin layer chromatography (tlc) on aluminum-backed silica plates as reported in our previous studies [21], [24]. Lipid spots corresponding to major lipid classes were scraped, counted and used to calculate radioactivity in each lipid class [21], [24].

#### Measurement of oxidative stress markers

Multiple tests were used to examine oxidative stress markers in the heart. First, cardiac content of lipid peroxidation products was measured using the LPO- test kit (Cayman, Ann Arbor, MI). Second, the amount of isoprostanes was quantified using gas chromatographic/negative ion chemical ionization mass spectrometry as described above for plasma samples. Finally, the contents of oxidized (GSSG) and reduced (GSH) glutathione in heart homogenates were determined by kits (Cayman) based on glutathione reductase coupled enzymatic recycling assay, and the ratio GSSH-to-GSH was calculated.

### Isolation and treatment of adult cardiomyocytes

Cardiomyocytes were isolated using a Langendorff perfusion system as described previously [24]. Briefly, hearts were perfused with Ca^2+^-free KH buffer containing collagenase II (0.9 mg/ml) for 20 min to ensure tissue digestion. Then, ventricle tissue was minced and re-suspended in Ca^2+^-free KH. Subsequently, Ca^2+^ was reintroduced incrementally back to 1.2 mmol/l and cardiomyocytes were allowed to settle, and then washed and counted for subsequent experiments. Myocytes were either seeded onto laminin-coated coverslips for culture, or left in suspension and used within 2 hours of isolation to investigate metabolite uptake and oxidation. Aliquots of cell preparations were used for microscopic examination and experiments with tryptophan blue to check for rod shaped cells and viability. The proportions of viable cells tested before and after experiments were comparable, with about 89% of cells with a rod shape appearance and 87% of cells without tryptophan blue.

### Measurements of fatty acid uptake and oxidation in cardiomyocytes

Assessment of FA uptake and oxidation in freshly isolated cardiomyocytes was performed using ^14^C palmitate (PerkinElmer, Waltham) according to the procedure of Luiken et al [25] as detailed in our previous publications [24], [26]. Briefly, 0.5 mmol/L [1-^14^C]-palmitate complexed to 0.3 mmol/L BSA in medium A supplemented with 1.0 mmol/L CaCl_2_ was added to cardiomyocytes pre-incubated in a capped glass vial in a 37°C shaking water bath (37°C). Incubations were continued for 3 minutes, a time within which palmitate oxidation was virtually null [25], and then cells were pelleted, washed and cell-associated radioactivity representing palmitate uptake was measured in beta counter [21]. Because CO_2_ is formed proportionally within the time period between 10 and 30 min after isotope addition, palmitate oxidation rate was measured at 30 min after addition of [1-^14^C]-palmitate (0.5 mmol/L) complexed to 0.3 mmol/L BSA and incubation at 37°C. The rate of [^14^C]-palmitate oxidation was measured as the sum of ^14^CO_2_, the final product of oxidation, and C^14^ labeled intermediate products according to our previous procedure [21]. Measurements were performed in triplicates, and blanks and controls were included for correction. Protein contents were measured and used to calculate rates of uptake [21].

### Assay of glucose uptake and oxidation

Glucose uptake rate was measured in cardiomyocyte suspension using 2-deoxy-D-[^3^H] glucose as described above for palmitate uptake with minor modifications [18]. To examine non-insulin dependent and insulin dependent uptake of glucose, cardiomyocytes were pre-incubated in medium A with or without 100 nM of insulin for 15 min at 37°C prior to the addition of tracers. Then, 2-deoxy-[^3^H]-glucose (0.5 μCi/ml) diluted in medium A with CaCl_2_ (1.0 mmol/L), 2-deoxyglucose (5 mM) and BSA (0.1%) was added, and incubation was continued for 30 min. Radioactivity incorporated in cells was determined by liquid scintillation. Rates of glucose oxidation were evaluated by the addition of 0.4 mmol/L [U-^14^C]-glucose (PerkinElmer) and 30 minute incubation period. Radiolabeled CO_2_, trapped in ethanolamine/ethylene glycol (1:2 vol/vol) was counted, and the oxidation rate was calculated after correction with the specific activity of tracers [26].

### Investigation of reactive oxygen species in the heart

Evaluation of reactive oxygen species (ROS) producing activity in hearts was performed with two procedures: visualization with fluorescence staining in heart sections and quantification of hydrogen peroxides in heart homogenates. Cell-permeable dye dihydroethidium (DHE) (Invitrogen) was used to evaluate the presence of ROS in heart sections as described by Kuroda et al. [27]. After topical application of DHE solution and incubation at 37°C for 30 minutes, heart sections were examined by fluorescence microscopy under a 585 nm filter and images were acquired using Zeiss Camera at 25X magnification [21]. In the second procedure, the fluorescence of hydrogen peroxides, downstream products of superoxides, was detected in homogenates using the Amplex Red hydrogen peroxide assay kit (Invitrogen) according to the manufacturer instructions.

### NADPH-dependent superoxide assay

NADPH-dependent superoxide production was measured in heart homogenates with the lucigenin chemiluminescent assay as described in previous studies [27], [28]. Immediately before the experiment, electron donor NADPH (300 μM) and lucigenin (5 μM) were added from stock solutions, so that the final volume reaction was 300 μl. Each sample was incubated at 37°C for 20 min and the lucigenin-dependent light emission was detected in 96 well plates by a chemiluminescence plate reader (BioTek, Winooski, VT). The effects of the following agents, pre-incubated for 15 min prior to addition of NADPH, were used to assess potential sources of superoxide production: nitric oxide synthase inhibitor NG-nitro-l-arginine methyl ester (L-NAME, 100 μM), xanthine oxidase inhibitor oxypurinol (100 μM), complex I mitochondrial electron chain inhibitor rotenone (20 μM), flavoprotein inhibitor diphenyleneiodonium (DPI, 10 μM) and inhibitor of NADPH oxidase (Nox) apocynin (30 μM). At this low concentration, apocynin was used as an inhibitor of Nox, although not specific of one particular isoform and possesses some antioxidant proprieties [29], [30]. In addition, the superoxide scavenger superoxide dismutase (SOD, 200 U/mL) was used as a positive control to confirm the specificity of superoxide detection. Each condition was tested in triplicates and superoxide production was expressed as arbitrary light units after subtraction of background reading set as reactions without NADPH.

### Quantification of palmitate-induced ROS production in isolated cardiomyocytes

To evaluate the impact of CD36 expression on fatty acid-induced ROS production, cardiomyocytes cultured in laminin-coated plates were incubated in medium with or without palmitate complexed with albumin. To assess the involvement of Nox in palmitate-induced ROS production, we treated the cells with palmitate in presence of VAS2870, a cell permeable inhibitor of Nox. Palmitate (250 μM) complexed with albumin at a molar ratio 4:1 was added alone or with Nox inhibitor VAS2870 (50 μM) to cells cultured in glass laminin-coated 12-well culture dishes in minimum essential medium with Hank's balanced salt solution, supplemented with bovine serum albumin (1 mg/ml), penicillin-streptomycin (100 U/ml), and glutamine (2 mM) [31]. Culture of treated and untreated (control) cells was continued for 6h, a period after which ROS production were examined. Given the limitations of the procedures of ROS productions and to ensure the accuracy and specificity of measurements, we used two assays concomitantly as recommended [28], [32]. First, estimation of ROS production in cardiomyocytes was performed with membrane-permeable fluorescent probe 5-(6)-chloromethyl-2′, 7′-dichlorodihydrofluorescein diacetate (CM-H_2_DCF/DA) (Molecular probes, Invitrogen, CA) which reacts with oxidants from different sources without discrimination of the origin. The fluorescent product issued from this reaction is retained in the cell and is used to estimate total cell ROS production. After treatment, cardiomyocytes were incubated with medium containing 1 μM CM-H_2_DCFDA for 30 min at 37°C, then, exhaustively washed with PBS prior to fluorescence measurement using a fluorescence microplate reader (Biotek, Winooski, VT) with 495 nm excitation and 520 nm emission for fluorescence [33]. Fluorescence intensity was expressed in arbitrary units (a.u.) after background subtraction. In the second procedure, production of superoxide production in cell lysate was assessed by lucigenin-enhanced chemiluminescence as described above.

### Isolation of microsomal and cell membrane fractions

Hearts were homogenized in ice-cold Buffer A and supernatant, collected after centrifugation at 600 *g* for 5 min, was re-centrifuged at 12,000 *g* for 15 min to sediment mitochondria. The resulting supernatant was transferred into another tube and centrifuged for 20 min at 30,000 g at 4°C to separate membrane fraction in the pellet and cytosol fraction in the supernatant [34]. Both membrane and cytosol fractions were used for western blotting and detection of p47^phox^, p67^phox^, PKCα and PKCδ.

### Immunoblotting and protein determination

Tissue proteins were analyzed by Western blotting as previously described [35] and the following primary antibodies were applied: phospho(Ser473)-Akt, Akt, PKCα, PKCδ, IRS1, phospho(Tyr608)-IRS-1, p47^phox^, p67^phox^, p22^phox^, Nox2 (also called gp91^phox^), Nox4, CD36, FATP1, PPARα and heart FABP (h-FABP). The specificity and reproducibility of these antibodies were validated prior to this study [16], [21], [24], [26], [36]. The list of antibodies is reported in Supplementary data (S2 Table). For total IRS1 and phosphorylated-IRS1, immunoprecipitation with protein G-agarose beads prior to immunoblotting. Band intensity for each protein was analyzed by densitometry (ImageJ version 1.37), and corrections were made using β-actin intensity reading [35].

### Gene Expression and qPCR

Tissue RNA extraction and synthesis of complementary DNA was performed as described in our previous procedure [35]. Quantitative polymerase chain reaction (qPCR) was performed using SYBR Green Supermix with iTaqDNA polymerase on the IQ5 thermocycler, and specifically designed and optimized oligonucleotides [35]. The sequence of the oligonucleotides is reported in Supplementary data (S1 Table). Data of qPCR were obtained as CT values, defined as the threshold cycle of PCR where products amplify exponentially. Difference in the CT values (ΔCT) was derived from the specific gene tested and CT of the control gene (β-actin) according to the equation 2^[CTactin − CTtarget gene]^ as described previously [36].

### Statistical analysis

Averaged values are presented as means ± SEM. Statistical significance was performed by One-way ANOVA test followed by Tukey’s test using GraphPad Prism 4 software (GraphPad Software). Statistical significance is recognized at p < 0.05

## Results

### Plasma and metabolic features of mice

**Fig 1:**
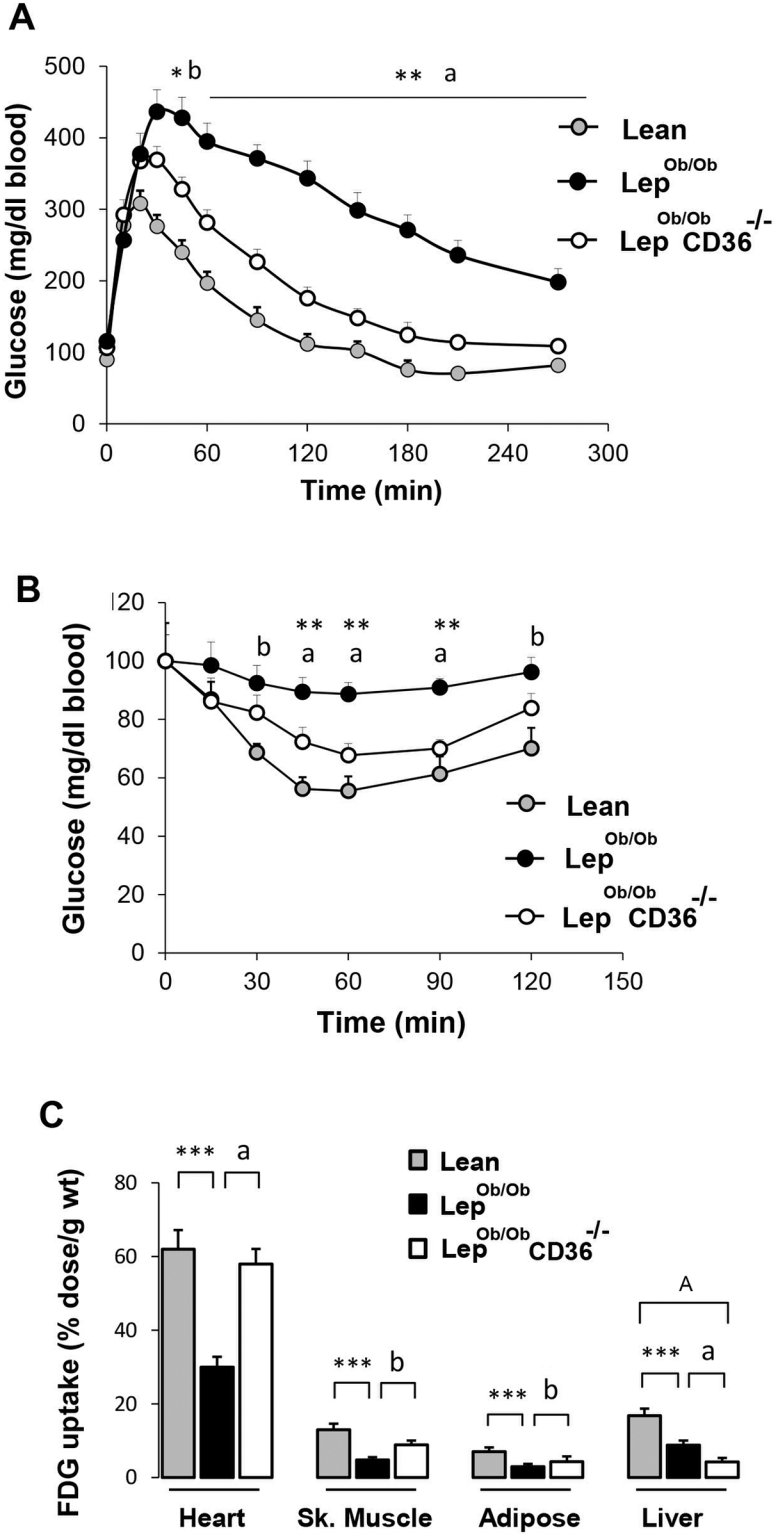
Effects of CD36 deficiency on glucose metabolism parameters. A) Glucose tolerance test (GTT) in overnight fasted control lean (Lean), leptin null (Lep^ob/ob^) and leptin and CD36 double null (Lep^ob/ob^CD36^-/-^) mice. B) Insulin tolerance test (ITT) in 4-h fasted mice. C) Uptake of ^18^F-2-FDG in organs of overnight fasted mice. Mice were injected with 5 μCi of ^18^F-2-FDG in a lateral tail vein and glucose uptake in indicated organ was determined as described in the Methods. Results are presented as mean ± SEM (n = 5–7 per group). For GTT and ITT, statistical differences between initial time (0 min) and subsequent time points (after glucose and insulin injection) were performed by repeated measurement ANOVA test. Differences between groups Tukey’s and student t tests. Statistical significance between Lep^ob/ob^ and Lean mice are indicated with asterisks ** p < 0.01, and * p < 0.05. Significance between Lep^ob/ob^ CD36^-/-^ and Lep^ob/ob^ mice are indicated with alphabetic letters with ^a^ p < 0.001, ^b^ p < 0.01 and ^b^ p < 0.05. Statistical significance between Lep^ob/ob^ CD36^-/-^ and Lean mice are indicated with a capital alphabetic letter ^A^ p < 0.01.

Body weight of Lep^ob/ob^CD36^-/-^ mice was reduced (-25%) compared to Lep^ob/ob^ mice but remained significantly (p < 0.001) higher than the weights of control Lean mice (Table 1). Heart weights were significantly higher (+21%) in Lep^ob/ob^ mice than lean controls and were modestly reduced in Lep^ob/ob^CD36^-/-^ mice. Compared to Lep^ob/ob^ mice, plasma FFA and TG levels were approximately 90 and 74%, respectively, greater in Lep^ob/ob^CD36^-/-^mice. The concentration of oxidative stress markers isoprostanes was increased in plasma Lep^Ob/Ob^ mice and was reduced in plasma of Lep^ob/ob^CD36^-/-^mice. Fasting plasma insulin concentration was strongly reduced and glucose fell to normal level in Lep^ob/ob^CD36^-/-^mice (Table 1). These data are consistent with increased insulin sensitivity in Lep^Ob/Ob^CD36^-/-^ mice as revealed by faster blood glucose clearance (GTT) (Fig 1A) and increased insulin sensitivity test (ITT) (Fig 1B).

**Table 1:** Body and organ weights, and plasma parameters for control lean (Lean), leptin null (Lep^ob/ob^) and leptin CD36 double null (Lep^ob/ob^CD36^-/-^) mice. Data are expressed as mean ± SEM with n = 12 per group.

	Lean	Lep^ob/ob^	Lep^ob/ob^CD36^-/-^	*p*	*p*
				Lep^ob/ob^ vs Lean	Lep^ob/ob^CD36^-/-^ vs Lep^ob/ob^
**Body weight (g)**	32.1 ± 2.1	69.3 ± 3.7	52.2 ± 2.6	0.001	0.05
**Heart Weight (mg)**	121 ± 3	147 ± 6	130 ± 5	0.05	NS
**Fatty acids (mM)**	0.65 ± 0.12	0.74 ± 0.14	1.41 ± 0.10	NS	0.001
**Triglycerides (mg/dl)**	72 ± 7	103 ± 12	169 ± 11	0.05	0.01
**8-Isoprostanes (pg/ml)**	178 ± 11	483 ± 19	209 ± 14	0.01	0.01
**Glucose (mg/dl)**	91 ± 5	124 ± 6	101 ± 8	0.05	NS
**Insulin (ng/ml)**	0.45 ± 0.12	5.98 ± 0.14	0.82 ± 0.37	0.001	0.001

### CD36 deficiency increased insulin sensitivity and glucose uptake in the heart

**Fig 2:**
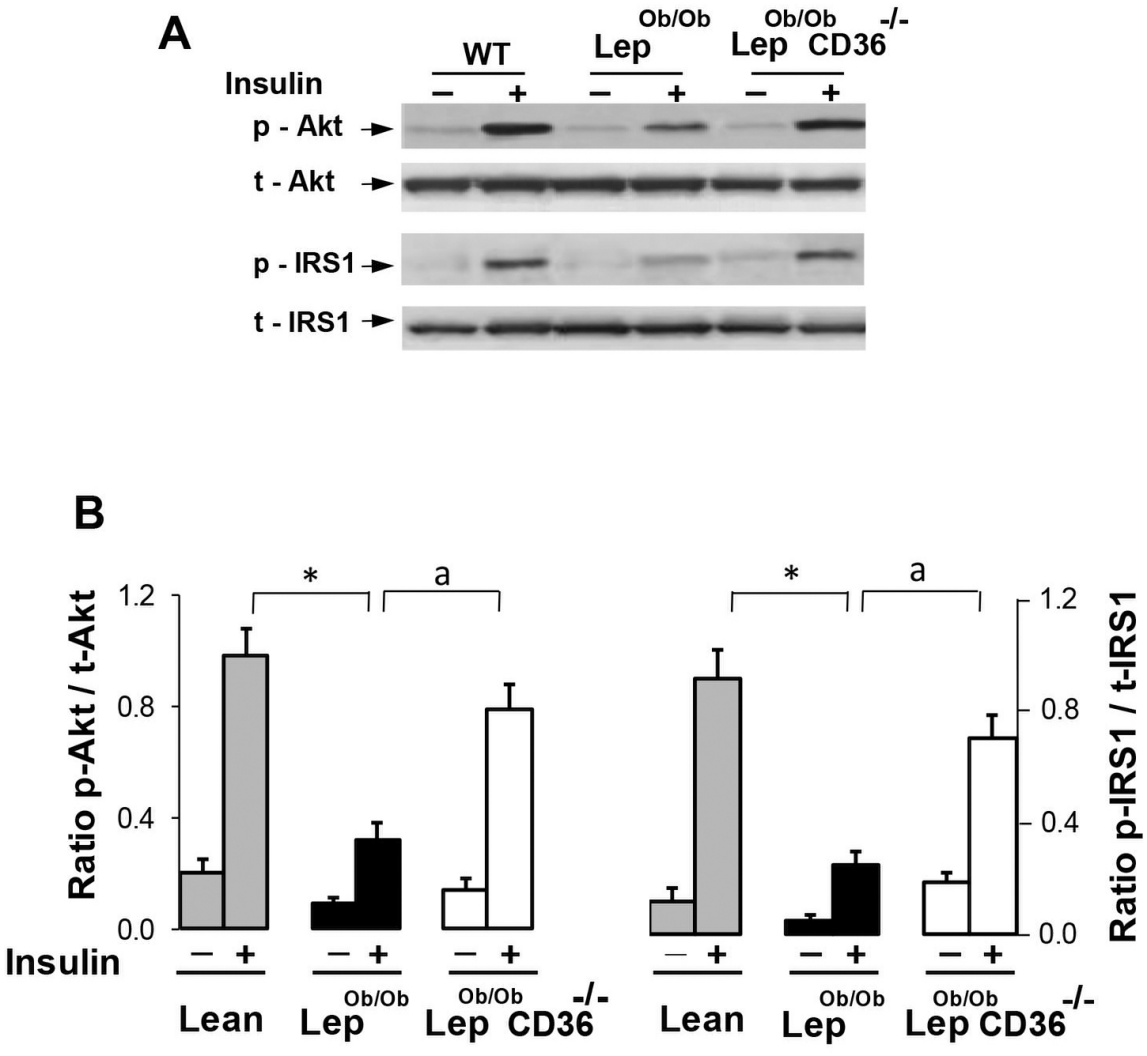
Effects of CD36 deficiency on insulin signaling. A) Representative blots and B) ratio of phosphorylated (p) to total (t) Akt and IRS1 in hearts 10 minutes after insulin injection. Results are presented as mean ± SEM (n = 5 per group). Statistical differences between Lep^ob/ob^ and Lean mice are indicated with an asterisk * p < 0.01, and differences between Lep^ob/ob^ CD36^-/-^ and Lep^ob/ob^ mice are indicated with an alphabetic letter with ^a^ p <0.05.

In light of the results of GTT and ITT presented above, we questioned if CD36 deficiency alters glucose uptake and insulin sensitivity in obese Lep^ob/ob^ mice. Glucose uptake assessed by ^18^F-2-FDG showed a reduction of about 2.2 fold in hearts of Lep^ob/ob^ mice compared to Lean mice, and marked increase in hearts of Lep^ob/ob^CD36^-/-^mice (Fig 1C). CD36 deficiency also enhanced glucose uptake in skeletal muscle (+61%) and adipose tissue (+32%), but reduced hepatic glucose uptake. In agreement with the robust increase of cardiac glucose uptake, insulin signaling was significantly improved in the hearts of Lep^ob/ob^CD36^-/-^ mice as revealed by increased Akt and IRS1 phosphorylation in response to insulin (Fig 2).

### CD36 deficiency decreased cardiac steatosis and reduced fatty acid uptake in the heart

**Fig 3:**
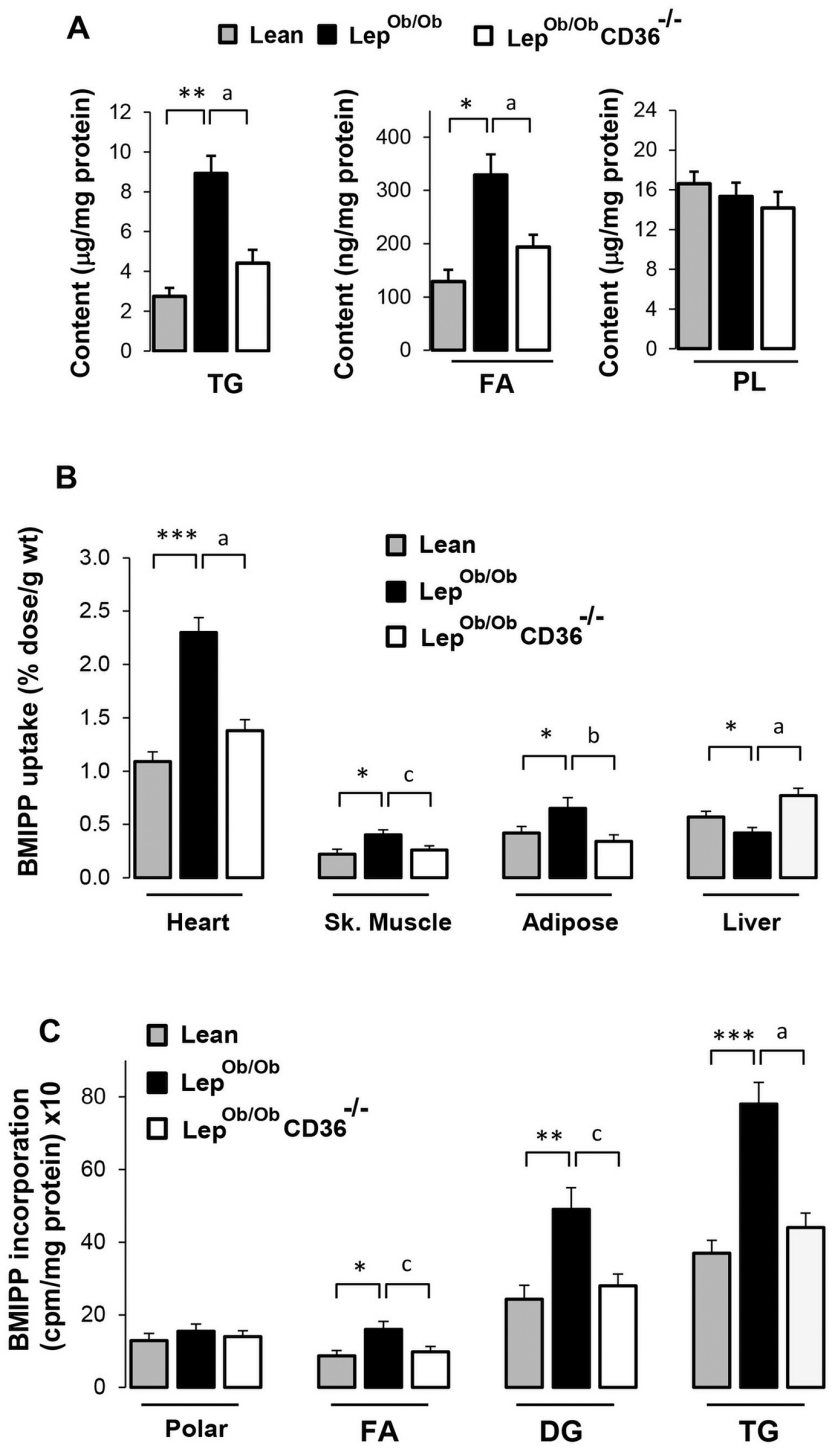
Effects of CD36 deficiency on cardiac lipid contents, fatty uptake and incorporation in lipids. Cardiac lipid contents (A) were determined enzymatically after lipid extraction as described in the Methods. Fatty acid uptake in indicated organs (B) was assessed using ^125^I-BMIPP. Mice were injected with 5 μCi of ^125^I-BMIPP into the tail vein and tissues were removed 2 h later. Incorporation of BMIPP in heart lipids (C) was examined after extraction and TLC separation. Polar lipids include phospholipids and monoacylglycerides. Results are presented as mean ± SEM (n = 6–7 per group). Differences between Lep^ob/ob^ and Lean mice are indicated with asterisks ** p < 0.01, and * p < 0.05. Differences between Lep^ob/ob^ CD36^-/-^ and Lep^ob/ob^ mice are indicated with alphabetic letters with ^a^ p < 0.01 and ^b^ p < 0.05.

Compared to Lean mice, the contents of triglycerides (TG) and free fatty acids (FAs) were markedly higher in the hearts of Lep^ob/ob^ mice and were normalized in Lep^ob/ob^CD36^-/-^ mice (Fig 3A), while phospholipid (PL) content was not altered. To address the reasons for reduced heart lipids, we examined the uptake of albumin-bound FA *in vivo* using non-degradable fatty acid analogue^125^I-BMIPP. In Lep^ob/ob^ mice, the uptake of BMIPP was markedly increased in hearts (+56%) and to a lesser degree in skeletal muscle (+42%) and adipose tissue (+29%), but was reduced in the liver compared to Lean mice (Fig 3B). Silencing CD36 in Lep^ob/ob^ mice induced a significant reduction of FA uptake in the heart (-42% compared to Lep^ob/ob^ mice), skeletal muscles (-31%) and adipose tissue (-37%), but a rise in liver (+35%) (Fig 3B). Analysis of ^125^I-BMIPP distribution in cardiac lipids showed a significant increase of FA accumulation and incorporation into diglycerides (DG) (+51%) and TG (+76%) of Lep^ob/ob^ compared to Lean mice (Fig 3C), indicating that part of FA taken up by cardiomyocytes were channeled towards the esterification pathway thus increasing cardiac lipid content. Silencing CD36 in Lep^ob/ob^ mice significantly reduced the proportions of BMIPP recovered in FA (-31%), DG (-37%) and TG (-46%) fractions, a result which is consistent with reduced cardiac lipid contents (Fig 3A).

### CD36 deficiency induced a switch of substrate utilization in cardiomyocytes

**Fig 4:**
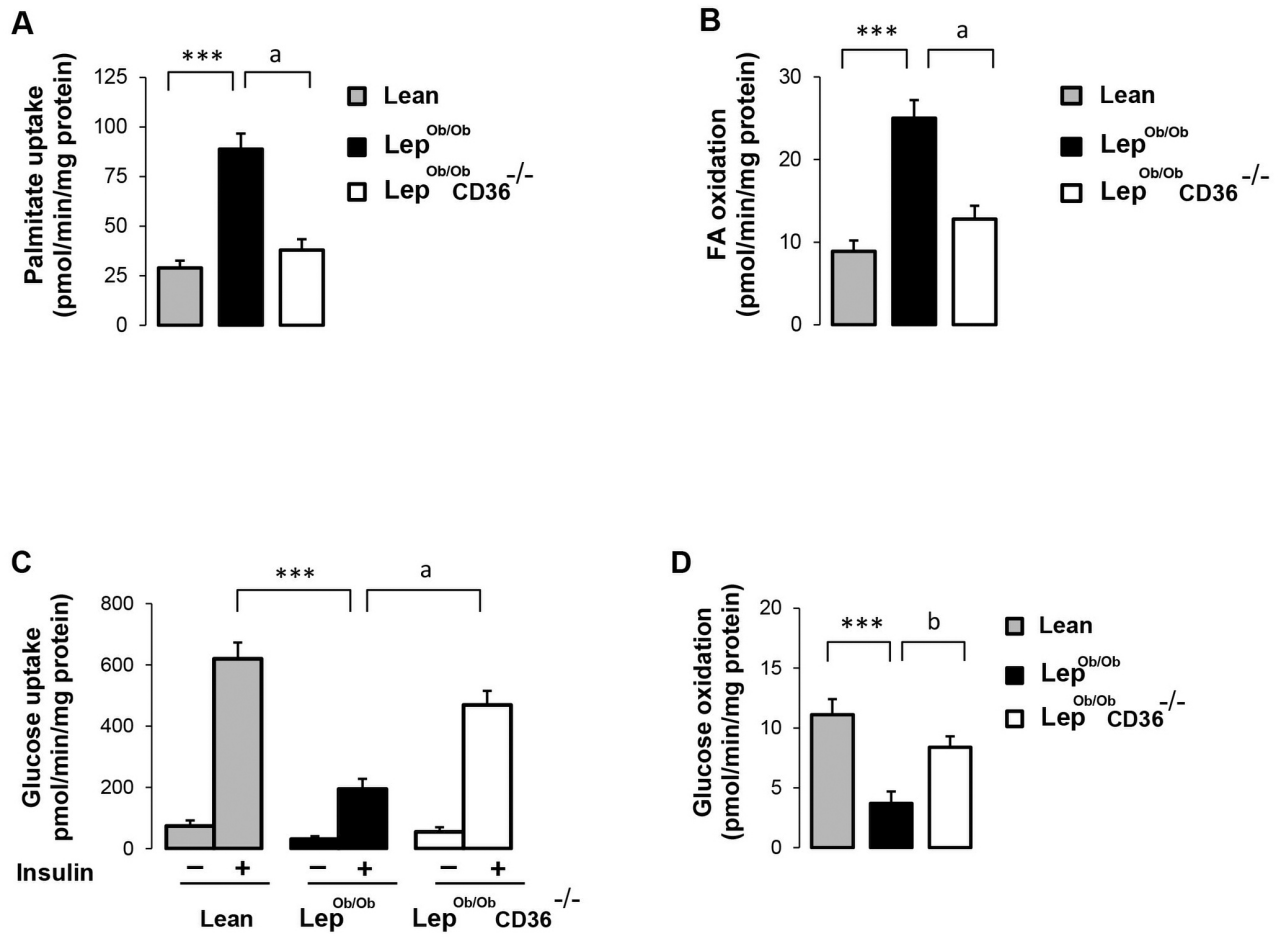
Uptake and oxidation of metabolites in cardiomyocytes. FA uptake (A) and oxidation (B) were determined in freshly isolated cardiomyocytes using [1-^14^C]-palmitate complexed to bovine serum albumin (BSA). Glucose uptake (C) was determined using 2-deoxy-D-[^3^H] glucose, and glucose oxidation (D) was examined with [U-^14^C]-glucose. Data are means ± SEM of triplicates from two different experiments. Differences between Lep^ob/ob^ and Lean mice are indicated with an asterisk * p < 0.01, and differences between Lep^ob/ob^ CD36^-/-^ and Lep^ob/ob^ mice are indicated with an alphabetic letter ^a^ p <0.05.

**Fig 5:**
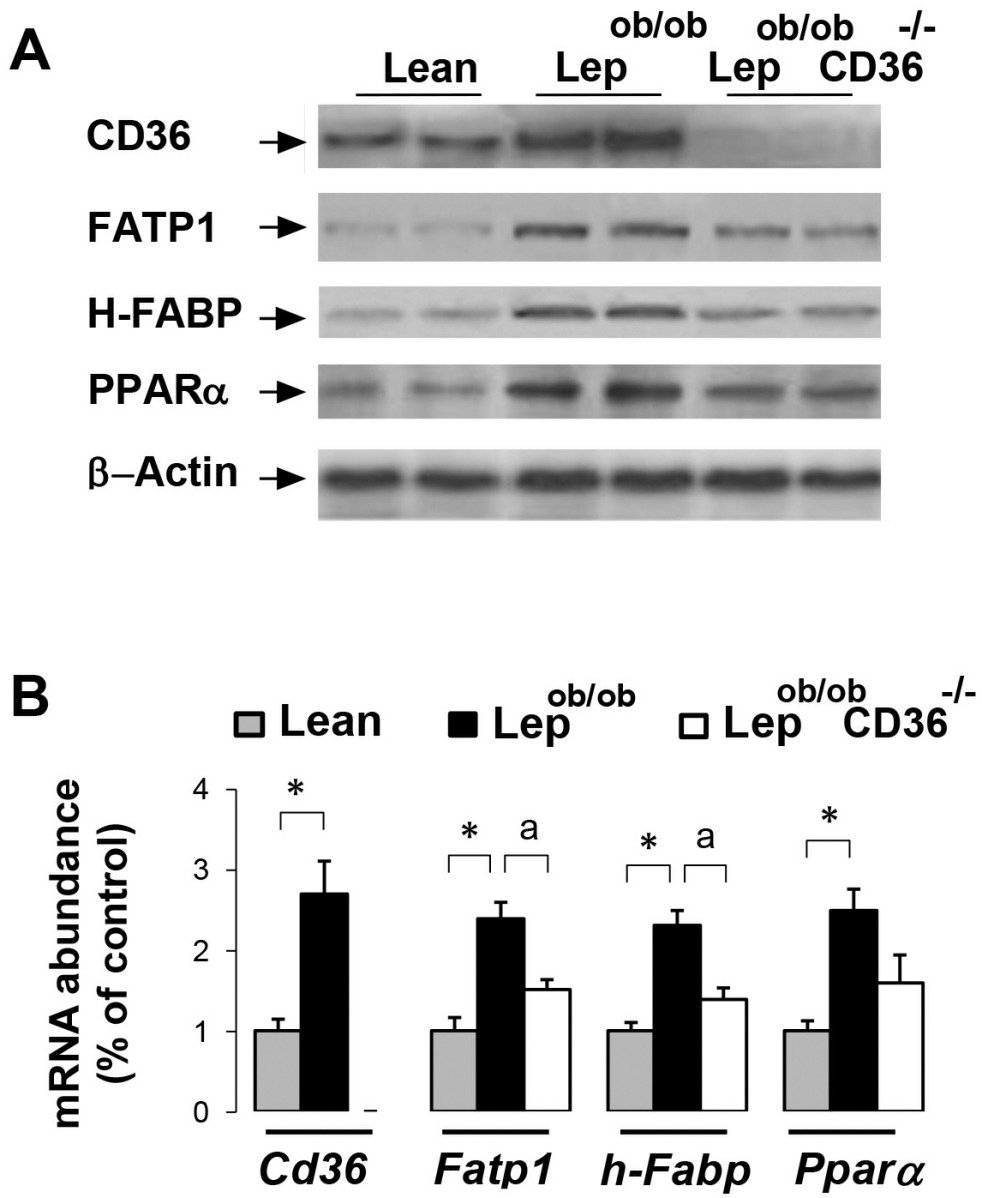
Effects of CD36 deficiency on protein expression. Representative blots (A) and mRNA abundance (B) of CD36, FATP1, H-FABP, PPARα in hearts of mice (n = 6 per group). Protein levels were examined by Western blotting and mRNA abundance was investigated with qPCR as described in the Methods. Differences between Lep^ob/ob^ and Lean mice are indicated with an asterisk * p < 0.05, and differences between Lep^ob/ob^ CD36^-/-^ and Lep^ob/ob^ mice are indicated with an alphabetic letter ^a^ p <0.05.

To examine whether alterations of glucose and FA uptake were associated with changes in substrate utilization, we examined metabolite oxidation in isolated cardiomyocytes. In agreement with the *in vivo* data, palmitate uptake in Lep^ob/ob^ cardiomyocytes was higher than Lean cardiomyocytes, but was clearly reduced in Lep^ob/ob^CD36^-/-^ cardiomyocytes (Fig 4A). Fatty acid oxidation paralleled the rate of uptake with higher oxidation in Lep^ob/ob^ cardiomyocytes and a reduction (-58%) in Lep^ob/ob^CD36^-/-^ cardiomyocytes (Fig 4B). Measurements of glucose uptake in isolated cardiomyocytes provided also similar results than the *in vivo* uptake of FDG (Fig 4C). Compared to Lean mice, insulin-stimulated deoxyglucose uptake was about 2.5 fold lower in Lep^ob/ob^ cardiomyocytes, and was markedly increased in Lep^ob/ob^CD36^-/-^ cardiomyocytes (+129% compared to Lep^ob/ob^ cardiomyocytes). By contrast to palmitate, the rate of glucose oxidation was lower in cells of Lep^ob/ob^ mice compared to Lean mice (-1.9 fold), but was increased (+1.3 fold) in cells of Lep^ob/ob^CD36^-/-^ mice (Fig 4D). These findings indicate that CD36 deficiency induced a switch of substrate utilization in favor of glucose. In agreement with these results, protein (Fig 5A) and mRNA (Fig 5B) levels of CD36, FATP1, H-FABP and PPARα were increased in Lep^ob/ob^ hearts compared to Lean hearts, and were relatively reduced in Lep^ob/ob^CD36^-/-^ hearts.

### CD36 deficiency reduced obesity-associated oxidative stress in the heart

**Fig 6:**
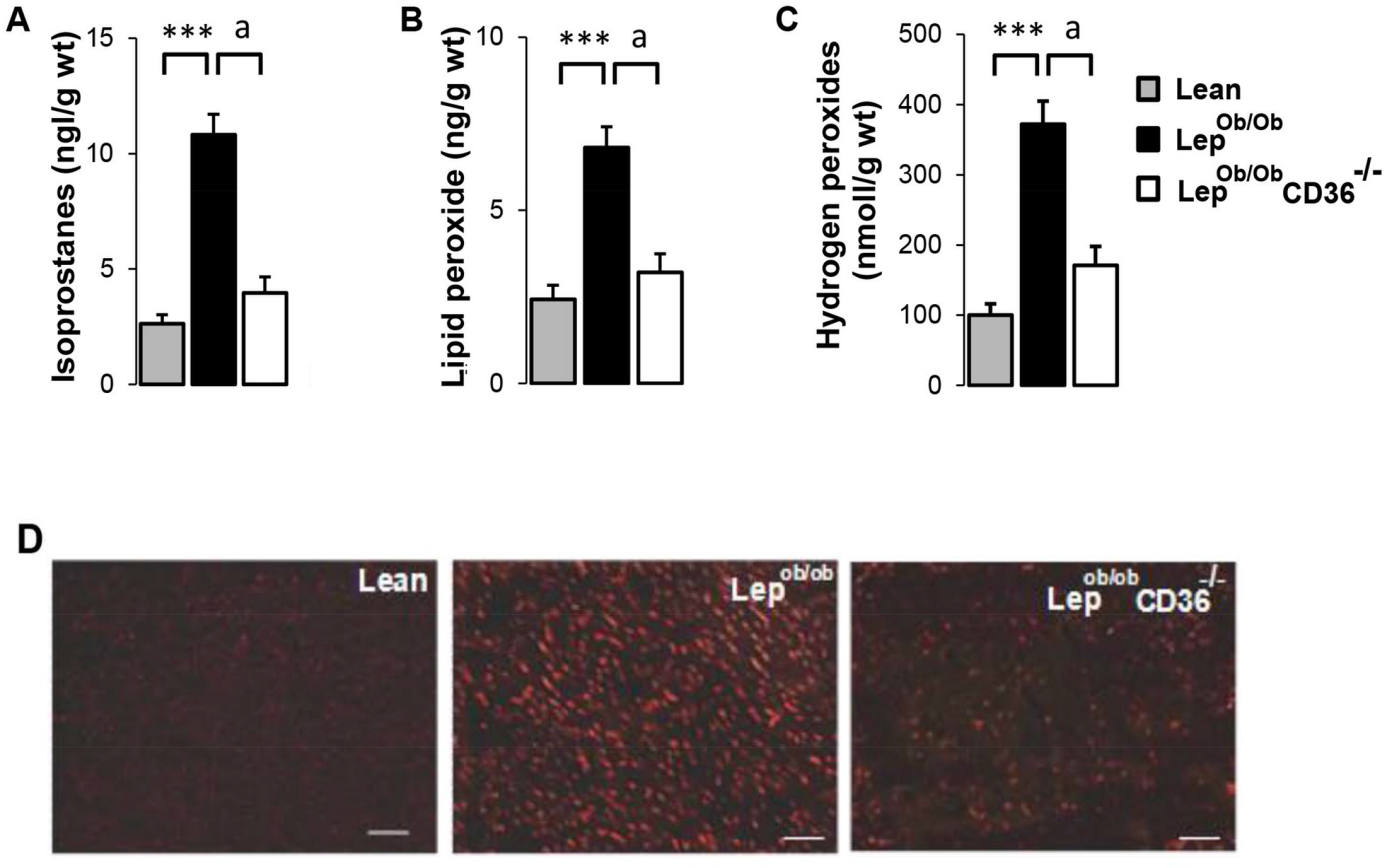
Effects of CD36 expression on cardiac oxidative stress markers. Contents of Isoprostanes (A) and lipid peroxides (B) were measured in heart homogenates using gas chromatographic/mass spectrometry and enzymatic kit, respectively. Hydrogen peroxides (C) were evaluated using Amplex Red method. Data are means ± SEM of duplicates from an n = 6 per group. Differences between Lep^ob/ob^ and Lean mice are indicated with asterisks ** p < 0.01, and differences between Lep^ob/ob^ CD36^-/-^ and Lep^ob/ob^ mice are indicated with alphabetic letters ^a^ p < 0.01 and ^b^ p < 0.05.

Steatosis is often associated with oxidative stress [1]. We thus examine cardiac content of isoprostanes and lipid peroxides, metabolites which serve as time-integrated markers of oxidative stress. Cardiac isoprostane (Fig 6A) and lipid peroxide (Fig 6B) contents were 2–4 fold higher in Lep^ob/ob^ mice than Lean mice, but were noticeably reduced in Lep^ob/ob^CD36^-/-^ mice. In agreement with these data, the ratio of reduced-to-oxidized glutathione (GSH-to-GSSG) was significantly reduced (p<0.01) in hearts of Lep^ob/ob^ mice (29.3 ± 1.5) compared to lean mice (45.4 ± 1.3), and was enhanced in hearts of Lep^ob/ob^CD36^-/-^ mice (38.1 ± 1.8, p< 0.05 compared to Lep^ob/ob^ mice) to a level close and not significantly different from Lean mice. These results are consistent with the measurements of 8-isoprostanes in the plasma shown in Table 1.

To further investigate the extent of oxidative stress, we evaluated hydrogen peroxide producing activity in heart homogenates by Amplex Red assay (Fig 6C). Cardiac content of hydrogen peroxides was about 3 fold higher in Lep^Ob/Ob^ mice than Lean mice, and was normalized in Lep^ob/ob^CD36^-/-^ mice (Fig 6C). These results are in accordance with fluorescent staining of superoxides in heart sections (Fig 6D) showing a distinctly higher intensity in hearts of Lep^ob/ob^ than Lean mice, and a marked reduction in hearts of Lep^ob/ob^CD36^-/-^ mice. These results indicate that CD36 deficiency reduced oxidative stress markers and ROS in the heart of Lep^Ob/Ob^ mice.

### CD36 regulates mitochondria- and NADPH oxidase-dependent superoxide production

**Fig 7:**
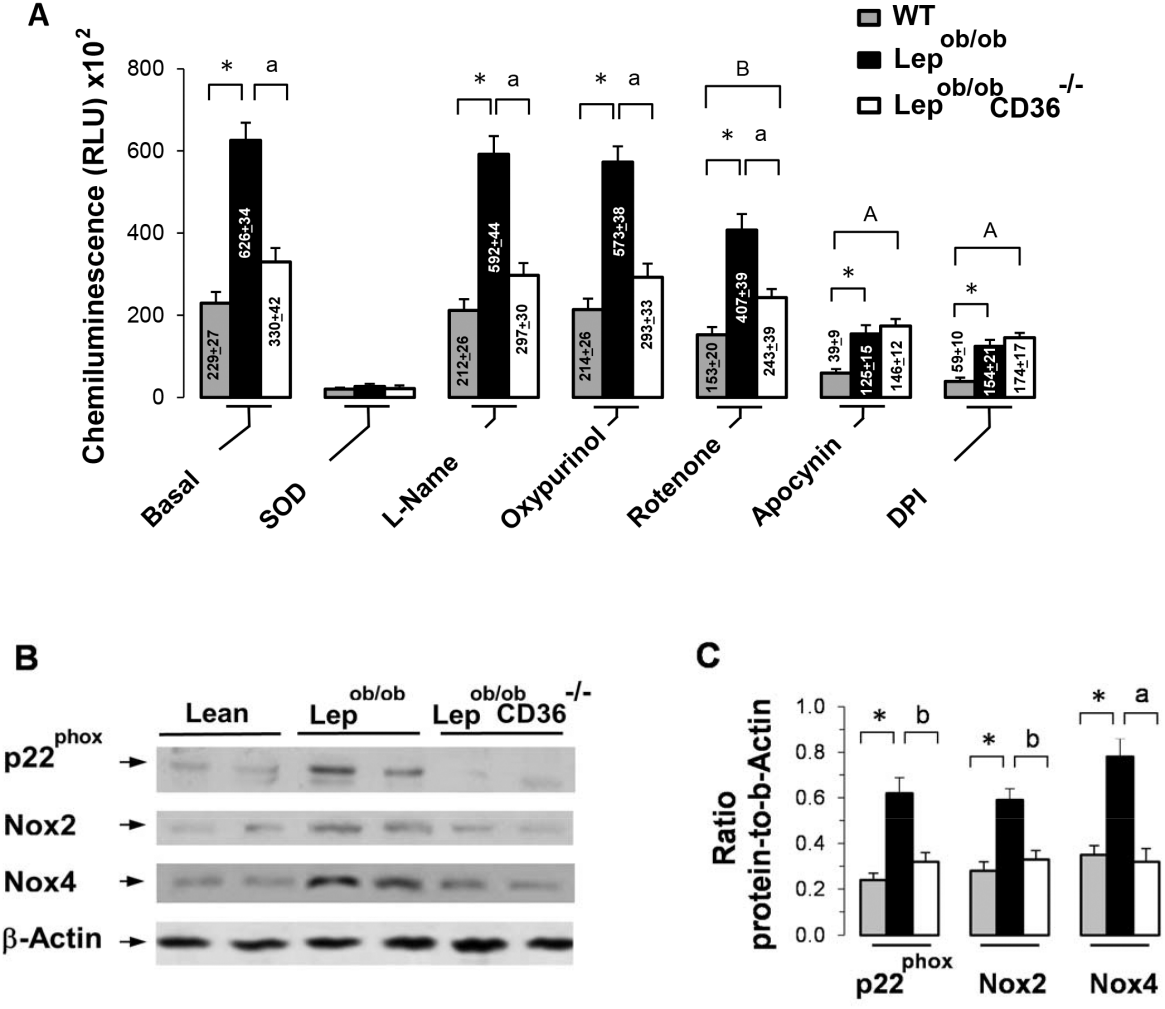
Effects of CD36 expression on NADPH-dependent superoxide production and expression of NADPH oxidase. A) NADPH-dependent and SOD-inhibitable superoxide was measured in heart homogenates by the lucigenin chemiluminescent method. Measurements were performed in preparations without inhibitors (basal) or in presence of superoxide dismutase (SOD) used to determine the specific of measurements, L-NAME (nitric oxide synthase inhibitor), Oxypurinol (oxidase inhibitor), Rotenone (complex I mitochondrial electron chain inhibitor), Apocynin (NADPH oxidase) and DPI (flavoprotein inhibitor). Measurements were conducted in triplicates from an n = 5 per group. B) Representative blots and C) ratio of p22^phox^, NADPH oxidase 2 (Nox2) and 4 (Nox4) to β-actin. Results are presented as Mean ± SEM and differences between Lep^ob/ob^ and Lean mice are indicated with asterisks with ** p < 0.01 and * p < 0.05. Differences between Lep^ob/ob^CD36^-/-^ and Lep^ob/ob^ mice are indicated with alphabetic letters ^a^ p < 0.01 and ^b^ p < 0.05. Differences between Lep^ob/ob^ CD36^-/-^ and Lean mice are indicated with A capital alphabetic letter ^A^ p < 0.05.

Several pathways are capable of generating ROS, some of which are associated with mitochondrial activity while others are linked to extra-mitochondrial enzymatic activities such as NADPH oxidase (Nox) and xanthine oxidase [37]. To examine the contribution of these pathways, we measured ROS production in heart homogenates using lucigenin chemiluminescent superoxide assay in the presence of inhibitors of Nox, nitric oxide synthase (NOS), xanthine oxidase and mitochondrial site I electron transport. Measurement without inhibitors (basal) provided total superoxides and shows that production was markedly higher in Lep^ob/ob^ than Lean mice, but was significantly lower in Lep^ob/ob^CD36^-/-^ hearts (Fig 7A). Compared to homogenates without inhibitors (basal), the addition of superoxide dismutase (SOD) strongly inhibited lucigenin signal in all groups (Fig 7A), thereby confirming that the signal measured in the assay was in fact superoxide-induced chemiluminescence. Neither NOS inhibitor L-NAME nor xanthine oxidase inhibitor oxypurinol had a significant effect on superoxide production. Mitochondrial inhibitor rotenone, however, reduced superoxides in Lean (-33% from basal), Lep^ob/ob^ (-34%) and Lep^ob/ob^CD36^-/-^ (-26%) hearts. Interestingly, Nox inhibitors apocynin and DPI reduced superoxide production in all groups, but the inhibitory effect was more pronounced in Lean mice (-82 and -74% from basal, respectively for apocynin and DPI) and Lep^ob/ob^ (-80 and -75%) mice than Lep^ob/ob^CD36^-/-^ mice (-55 and -47%) as shown in Fig 7A and S3 Table. These results indicate that both mitochondria and Nox were important sources of excess ROS production in the heart of Lep^ob/ob^ mice, and that CD36 deficiency mostly reduced Nox-dependent ROS production suggesting that CD36 is involved in the regulation of Nox activity.

### CD36 deficiency altered NADPH oxidase expression in the heart

To elucidate the role of CD36 in Nox regulation, we examined the expression of Nox2 and Nox4 isoforms, and the regulatory subunit unit p22^phox^. Western blot analysis showed that protein levels of Nox2, Nox4 and p22^phox^ were significantly increased in Lep^ob/ob^ mice, and were reduced in Lep^ob/ob^CD36^-/-^ mice (Fig 7B and 7C) (S1 and S2 Figs). The change of Nox2 and Nox4 expression were also reflected at the mRNA levels. However, the mRNA abundance of *Nox1* was comparable between groups as shown in supplementary data (S3 Fig).

### CD36 deficiency altered the distribution of p47^Phox^, p67^Phox^and PKC in cell membrane

**Fig 8:**
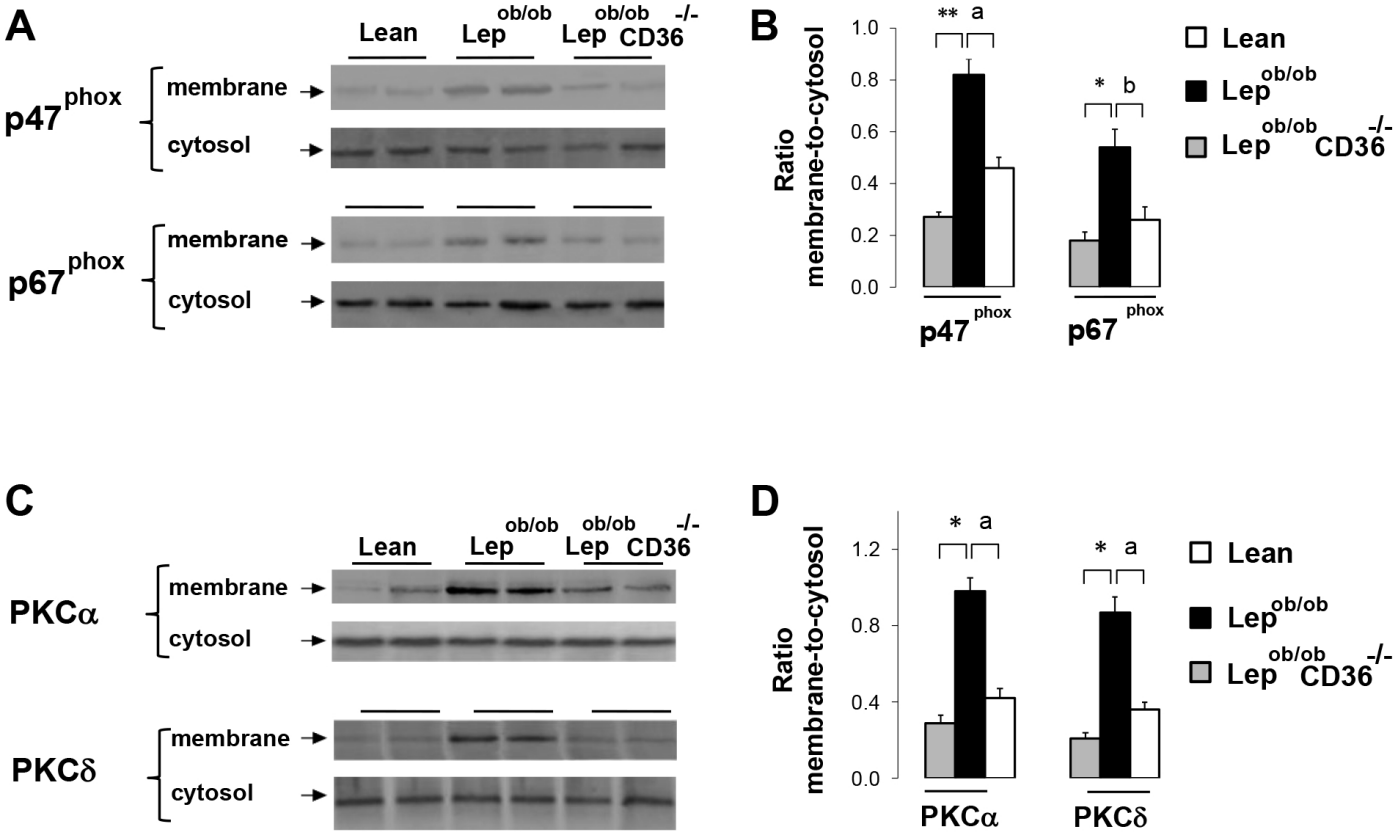
Effects of CD36 expression on the distribution of NADPH oxidase (Nox) and PKC in membrane and cytosol fractions. Representative blots (A and C) and means of membrane-to-cytosol ratios of optic density (B and D) of p47^phox^, p67^phox^, PKCα and PKCδ proteins. The procedures of separation of membrane and cytosol fractions and western blotting are described in the Methods. Differences between Lep^ob/ob^ and Lean mice are indicated with asterisks with ** p < 0.01 and * p < 0.05, and differences between Lep^ob/ob^ CD36^-/-^ and Lep^ob/ob^ mice are indicated with alphabetic letters ^a^ p < 0.01 and ^b^ p < 0.05.

The translocation of p47^Phox^ and p67^Phox^ subunits from the cytosol to cell membrane is required to form active Nox2 enzymatic complex. Therefore, the ratio cell membrane-to-cytosol is indicative of protein complex activation. Western blots revealed significant increase (p<0.05) in the ratio of cell membrane-to-cytosol of both p47^Phox^ and p67^Phox^ in Lep^ob/ob^ hearts, and a marked reduction in Lep^ob/ob^CD36^-/-^ hearts (Fig 8A and 8B). Given that PKC has been implicated in Nox activation [38], [39], we examined PKC distribution between cell membrane and cytosol. As shown in Fig 8C and 8D, membrane-to-cytosol ratios of PKCα and PKCδ were noticeably increased in hearts of Lep^ob/ob^ mice, but were significantly reduced (p<0.01) in Lep^ob/ob^CD36^-/-^ mice suggesting that CD36 deficiency reduced membrane translocation of PKCα and PKCδ in parallel to diminishing Nox activity.

### CD36 mediated palmitate-induced superoxide production

**Fig 9:**
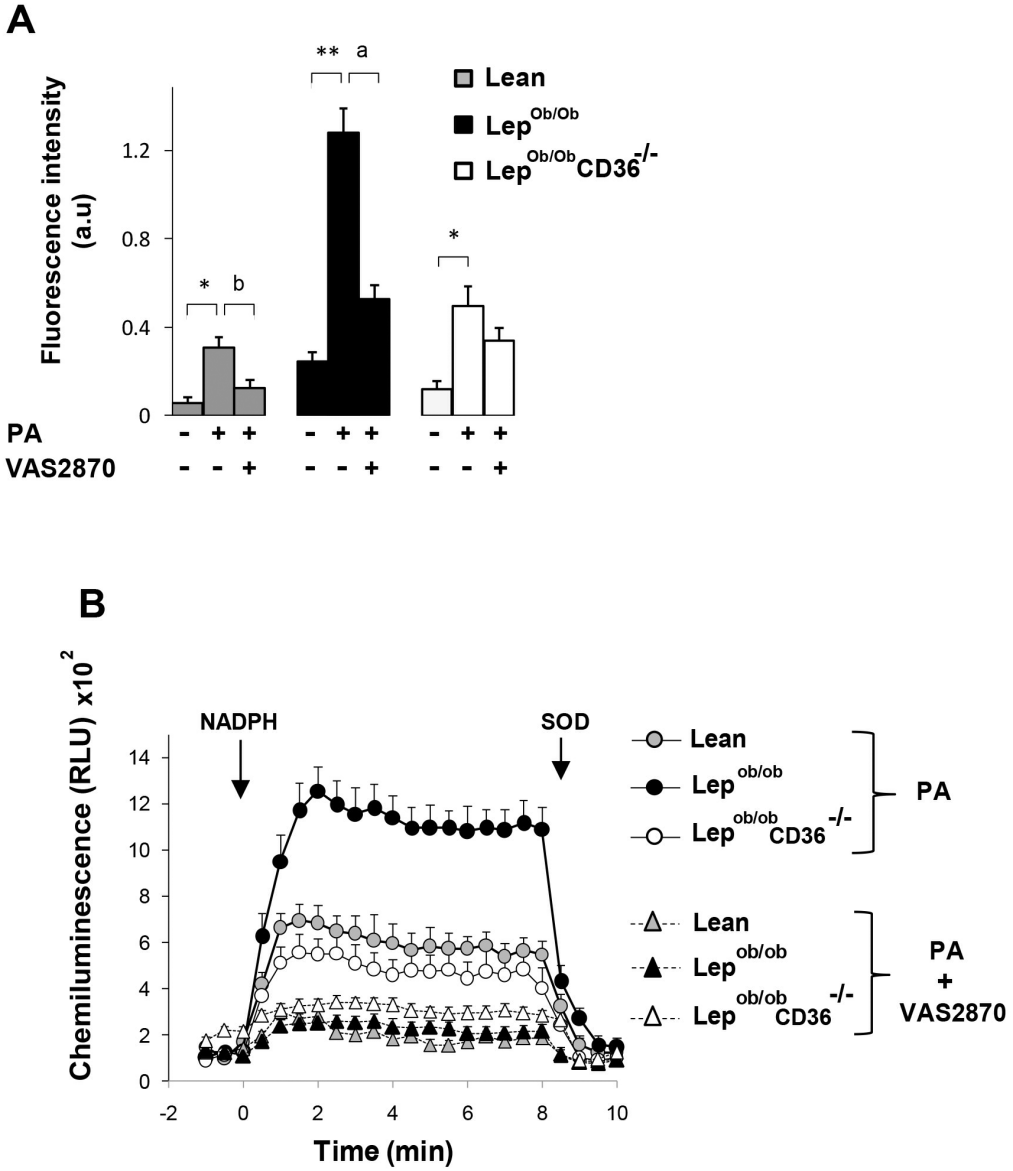
Effects of palmitate treatment on ROS production in cardiomyocytes. Quantification of ROS production with CM-H_2_DCF/DA (A) and lucigenin chemiluminescent method (B) in cardiomyocytes treated with palmitate for 6h in presence or absence of Nox inhibitor VAS2870. For measurement of superoxide production, cells were loaded with loaded with 0.5 μM CM-H_2_DCF/DA in PBS and fluorescence was recorded using a microplate reader with 530nm emission and 485nm excitation. Background fluorescence was measured in control cells without dye loading and subtracted from all data. Measurements of superoxide production with lucigenin chemiluminescent method are performed as described in the Method section. Data are means ± SEM of triplicates from an n = 4 mice per group. Differences between untreated and palmitate-treated cells of the same genotype are indicated with asterisks ** p < 0.01 and * p < 0.05. Differences between palmitate-treated and palmitate-VAS2870-treated cells of the same genotype are indicated with alphabetic letters ^a^ p < 0.01 and ^b^ p < 0.05.

To further gain information about the role of CD36 and Nox in cardiac oxidative stress, we examined ROS production in isolated cardiomyocytes cultured with palmitate in presence or absence of cell permeable Nox inhibitor VAS2870. ROS production was first assessed with CM-H_2_DCF/DA dye which reacts with ROS from all sources leading to the formation of fluorescent oxidation products. In basal cardiomyocytes cultures (no palmiate and no VAS2870), fluorescence intensity was higher in Lep^ob/ob^ cells compared to lean and Lep^ob/ob^CD36^-/-^ cells (Fig 9A). Palmitate treatment induced a stronger increase of fluorescence intensity in Lep^ob/ob^ cells (+ 3.6 fold above non-treated cells) than Lean and Lep^ob/ob^CD36^-/-^ cells. The presence of Nox inhibitor reduced palmitate-elicited fluorescence in cardiomyocytes of Lep^ob/ob^ (-59%) and lean mice (-40%), but was less efficient in cardiomyocytes of Lep^ob/ob^CD36^-/-^mice (Fig 9A) (S4 Table). Having shown that palmitate-induced ROS production was dependent on CD36 expression and was reduced by VAS2870, we sought to confirm these results by measuring SOD-inhibitable superoxide production using lucigenin chemiluminescence. As shown in Fig 9B, palmitate overload induced a strong induction of NADPH-induced superoxide production in cardiomyocytes of Lep^ob/ob^ mice, but lower induction in cardiomyocytes of Lean and Lep^ob/ob^CD36^-/-^ mice. Following the addition of NADPH, the maximum signals was approximately 2 fold higher in Lep^ob/ob^ than in Lean and Lep^ob/ob^CD36^-/-^ cardiomyocytes. The addition of superoxide dismutase (SOD) strongly inhibited lucigenin signal in all groups, thereby confirming that the signal measured in the assay was superoxide-induced chemiluminescence. Treatment with VAS2870 reduced palmitate-induced superoxide production in all groups but the strongest inhibitory effect was in Lep^ob/ob^ cardiomyocytes (-3 fold compared to palmitate alone) and the lowest effect was in Lep^ob/ob^CD36^-/-^ cardiomyocytes. The inhibitory effect of VAS2870 on superoxide production shown in these experiments corroborate the results of apocynin presented earlier in Fig 7A for total heart.

## Discussion

Obesity-associated cardiac steatosis induces lipotoxicity leading to insulin resistance and metabolic dysfunctions. Whereas increased availability of blood lipids is implicated in cardiac steatosis, the mechanisms by which FFAs induce lipotoxicity are not fully understood. With this in mind, we aimed to explore the role of CD36 in obesity-induced cardiac steatosis and lipotoxicity in the Lep^ob/ob^ mice model. The results reported here indicate (1) that cardiac steatosis in Lep^ob/ob^ mice is associated with increased CD36 expression in conjunction with enhanced oxidative stress, (2) show that the induction of Nox activity is an important component in CD36-induced ROS production, and (3) highlight the impact of CD36 deficiency in cardiac oxidative stress and lipotoxicity.

Previous studies by others and us have shown that CD36 deficiency in lean C57BL/6J background mice reduced the uptake of fatty acids in peripheral organs while improving insulin sensitivity and glucose disposal [17], [18], [19]. The objective of the present study was to examine the impact of CD36 deficiency on obesity-associated oxidative stress and lipotoxicity in heart. According, studies were conducted in Lep^ob/ob^ and Lep^ob/ob^ CD36^-/-^ mice, while using lean control mice only as a reference. As previously reported [5] [40], adult Lep^ob/ob^ mice displayed severe obesity with increased heart weight and lipid infiltration. Disruption of CD36 expression markedly reduced cardiac lipid content, but induced a modest reduction of heart weight. The causal relationship between steatosis and hypertrophy in heart has been examined in multiple animal models and remains uncertain [6], [9]. This may be related to the fact that the pathogenesis of cardiac hypertrophy is complex, involving multiple factors that extend far beyond lipid infiltration [9]. Although cardiac lipid content was reduced, blood lipid levels were rather increased in Lep^ob/ob^CD36^-/-^ mice. These results are consistent with previous investigations in lean CD36^-/-^ mice [16], [18], [19] showing that reduction of intravascular lipolysis of TG-rich lipoprotein and decreased uptake of fatty acids in peripheral tissues are the main reasons for the enlargement of circulating lipid pool.

The finding that CD36 expression is increased in the heart of Lep^ob/ob^ mice is consistent with previous findings in genetically and diet-induced obese mice [40], [41], [42]. The availability of FAs is also increased in Lep^ob/ob^ mice creating a mismatch between delivery and utilization rates of FAs, and leading eventually to excess lipid accumulation. In addition to reducing cardiac steatosis, CD36 deficiency resulted in a marked reduction of FA utilization and simultaneous increase of glucose uptake and oxidation. This shift of cardiac substrate utilization from FA to glucose was associated with a marked activation of insulin signaling as indicated by increased phosphorylation of insulin signaling proteins (Fig 2). These findings are in line with prior investigations showing that the heart is well equipped to compensate for reduced lipid oxidation by increasing the utilization of other metabolites including glucose [3], [9], [43]. Currently, there is no finding to directly implicate CD36 in insulin signaling pathway but one possible mechanism could be linked to the role of CD36 in lipid homeostasis. It is possible that CD36 deficiency improves insulin sensitivity in the Lep^ob/ob^ heart indirectly though the reduction of toxic lipid accumulation known to hinder insulin signaling [14], [26]. In support of this hypothesis, a strong association between insulin resistance and increased cardiac lipid accumulation has been reported in obese rats [44], [45] and genetically altered mice [46], [47].

While some studies consider mitochondria as a major site for superoxide production caused by electron leakage from the oxidative phosphorylation pathway [11], others implicate extra-mitochondrial enzymes, especially Nox in ROS production [29], [37], [48], [49]. In the present study, we show that mitochondrial and extra-mitochondrial ROS production are both enhanced in hearts of obese Lep^ob/ob^ mice; however the increase in the latter pathways was stronger. These findings are in line with previous studies reporting that Nox activity/expression is increased in mice with genetic [1], [34], [50], [51] or diet-induced obesity [52]. Our studies further provide a strong link between CD36 expression and Nox-dependent superoxide production, such that silencing CD36 reduced Nox expression and abrogated excess production of ROS in the heart (Figs 6 and 7) and isolated cardiomyocytes (Fig 9). Besides cardiomyocytes, Nox isoforms are also expressed in several cell types of heart such as monocytes, macrophages and microvascular endothelial cells [28], [48], [53], [54], and are upregulated by insulin resistance [50], [55] and obesity [50], [56]. Therefore, we cannot exclude the possibility that multiple cell types contribute collectively to increase ROS production in the heart of Lep^ob/ob^ mice.

Our results show that CD36 deficiency strongly reduced palmitate induction of Nox-dependent ROS production (Fig 9). These findings provide evidence of regulatory mechanisms between CD36 expression and Nox-dependent ROS production in cardiomyocytes which may explain, at least in part, lower oxidative stress markers in total heart (Fig 7A). Moreover, the inhibitory effect of VAS2870 on palmitate-induced ROS production is the strongest in cardiomyocytes of Lep^ob/ob^ mice in which CD36 expression is the highest. These results are consistent with the effects induced by apocynin in Lep^ob/ob^ hearts and suggest that Nox-dependent superoxide production is regulated by CD36 expression. Although, VAS2870 is known as a selective inhibitor of Nox, its action is not specific to a single isoform [29]. Therefore, measurements of superoxide production in cardiomyocytes most likely represent the action of VAS2870 on multiple isoforms without the distinction of a specific isoform contribution. In general, two mechanisms could be involved in the activation of Nox: chronic increase in the expression and acute increase in oxidase complex formation secondary to posttranslational modification of regulatory subunitsp47^phox^ and p67^phox^ [34], [52]. Given that CD36 deficiency reduced protein abundance of Nox and translocation of regulatory units (Figs 7 and 8), we cannot exclude the possibility that both acute and chronic activation of Nox are modulated by CD36 expression.

Another important finding of this study is that CD36 deficiency reduced the incorporation of FA into DG and TG (Fig 3C). These changes were associated with a reduction of cell membrane-associated PKC, Nox activity and ROS production (Figs 7 and 9). Although the chain of events induced by CD36 deficiency is still not clear, our results provide evidence that CD36 expression plays an important role in regulating the utilization of cardiac lipids and activation of PKC and Nox. Excessive supply of FAs and increased intracellular lipids has been implicated in the activation of both Nox and PKC [39], [56], [57], [58], [59]. There is also evidence in the literature to indicate the existence of regulatory mechanisms between cellular lipids, PKC and Nox. In fact, DG is a well-known signal for PKC activation [60], [61], and both DG and FA act synergically to increase PKC translocation [61]. Moreover, activation of PKC increases the translocation of p47^phox^ regulatory subunit and formation of active heterodimer Nox complexes [38], [39], [59], [62]. It is thus possible that reduction of intracellular lipid mediators is among the early steps through which CD36 deficiency reduces PKC and Nox activation. Translocation p47^phox^ and p67^phox^ are mostly relevant to the activation of Nox2, while p22^phox^ regulates Nox4 activation [37], [63]. Therefore, it is possible that CD36 acts through different pathways to regulate Nox2 and Nox4 activity. Of note, multiple mechanisms of regulation of Nox have been described, some of which are isoform-specific [37], [63], and may also differ between phagocytic and non-phagocytic cells [64], [65]. Therefore, it is possible that multiple mechanisms are involved simultaneously to mediate CD36 regulation of Nox isoforms in the heart.

In conclusion, the current study provides evidence that CD36 plays an important role in cardiac steatosis and lipotoxicity in obese Lep^ob/ob^ mice. The mechanisms for this effect are linked to increased fatty acid influx that may trigger a cascade of events leading to the activation of NADPH oxidase, increased ROS production and insulin resistance.

## Supporting Information

S1 Fig Representative immunoblot of Nox2 protein. (PDF)

S2 Fig Representative immunoblot of Nox4 protein. (PDF)

S3 Fig Quantitative PCR (qPCR) analysis of Nox isoforms in the heart. (PDF)

S1 Table Primer sequences for quantitative polymerase chain reaction q-PCR. (PDF)

S2 Table List of primary antibodies used in the study. (PDF)

S3 Table Statistical analysis of data presented in Fig 7. (PDF)

S4 Table Statistical analysis. (PDF)

## Abbreviations

ROS: Reactive oxygen species
FDG: Fluorodeoxyglucose
BMIPP: β-methyl-p-123I- Iodophenyl-Pentadecanoic Acid
CM-H2DCF/DA: 5-(6)-chloromethyl-2′, 7′-dichlorodihydrofluorescein diacetate
DPI: Diphenyleneiodonium
qPCR: quantitative PCR
IRS-1: Insulin receptor substrate 1
FFA: Free fatty acid
TG: Triglyceride
GTT: Glucose tolerance test
ITT: Insulin tolerance test
LPL: Lipoprotein lipase
Nox: NADPH oxidase: Nicotinamide adenine dinucleotide phosphate (NADPH) oxidase
PKC: Protein kinase C
