# Supplementary material for: Cluster Differentiating 36 (CD36) Deficiency Attenuates Obesity-Associated Oxidative Stress in the Heart
Source: PLoS One. 2016 May 19;11(5):e0155611. doi: 10.1371/journal.pone.0155611 (PMC4873222; doi:10.1371/journal.pone.0155611)

SUPPLEMENTARY DATA – Figure 2:

Representative of immunoblot of Nox4. Equal amounts of proteins were separated by electrophoresis on SDS polyacrylamide gels, and blots were probed with Nox4 antibody. In each blot, heart protein samples were loaded in lanes numbered from 1 to 9 and protein markers with known molecular weights were layered in an adjacent lane. In this figure, lanes 1, 3 and 4 are samples of Lean mice, lane 2, 5 and 6 are samples of Lep$^{ob/ob}$ mice and samples 7-9 are samples of Lep$^{ob/ob}$ CD36$^{-/-}$ mice.

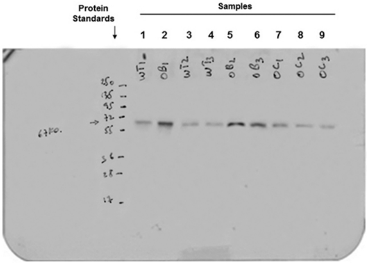

Supplement: S2 Fig — (PDF) [file pone.0155611.s002.pdf]
