# Supplementary material for: Cluster Differentiating 36 (CD36) Deficiency Attenuates Obesity-Associated Oxidative Stress in the Heart
Source: PLoS One. 2016 May 19;11(5):e0155611. doi: 10.1371/journal.pone.0155611 (PMC4873222; doi:10.1371/journal.pone.0155611)

SUPPLEMENTARY DATA – Figure 3

Quantitative PCR (qPCR) analysis of *Nox* isoforms in hearts of Lean, $Lep^{ob/ob}$ and $Lep^{ob/ob}CD36^{-/-}$ mice: qPCR analysis was performed using a Light Cycler 480 SYBR Green I Master and specific primers which sequences are reported in supplementary data - Table 1. Bars are means ± S.E.M. Difference between $Lep^{ob/ob}$ and Lean mice are indicated with an asterisk with * $p < 0.05$, and difference between $Lep^{ob/ob}$ and $Lep^{ob/ob}CD36^{-/-}$ mice are indicated with an alphabetic letter with ^{a} $p < 0.05$. NS = nonsignificant.

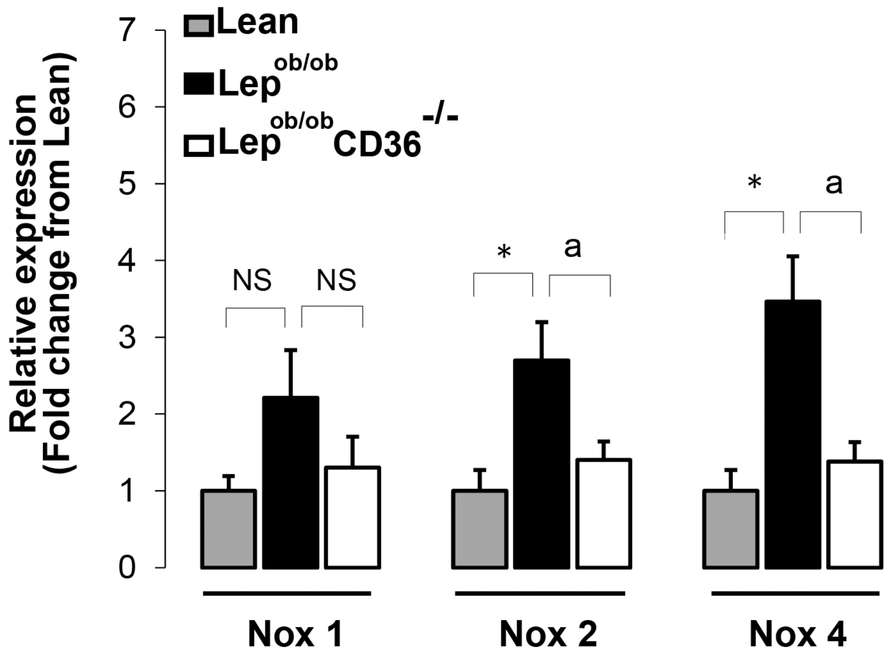

Supplement: S3 Fig — (PDF) [file pone.0155611.s003.pdf]
