# Supplementary material for: Cluster Differentiating 36 (CD36) Deficiency Attenuates Obesity-Associated Oxidative Stress in the Heart
Source: PLoS One. 2016 May 19;11(5):e0155611. doi: 10.1371/journal.pone.0155611 (PMC4873222; doi:10.1371/journal.pone.0155611)
Supplement: S1 Table — (PDF) [file pone.0155611.s004.pdf]

SUPPLEMENTARY DATA - Table 1

Primer sequences for quantitative polymerase chain reaction q-PCR.

**Gene**	**Encoding protein**	**Forward 5'-3'**	**Reverse 5'-3'**
cd36	CD36	CTGTTATTGGTGCAGTCCTGGC	TATGTGGTGCAGCTGCTACAGC
fatp1	FATP1	GTTGGCTGCTGTGTCCTG	GAACCGTGGATGAACCTAAG
h-fatp	Heart-FABP	AGTCACTGGTGACGCTGGACG	AGGCAGCATGGTGCTGAGCTG
pparα	PPARα	GGAGGCTAATAGGATTCAGACAG	AACAGACCGCTCAGACTTCG
β-actin	β-Actin	AGGGAAATCGTGCGTGACAT	CGTTGCCAATAGTGATGACC
nox1	NOX1	AATGCCCAGGATCGGGT	GATGGAAGCAAAGGGAGTGA
nox2	NOX2	CCCTTTGGTACAGCCAGTGAAGAT	CAATCCCGGCTCCCACTAACATCA
nox4	NOX4	GGATCACAGAAGGTCCCTAGCAG	GCGGCTACATGCACACCTGAGAA
