# Supplementary material for: Cluster Differentiating 36 (CD36) Deficiency Attenuates Obesity-Associated Oxidative Stress in the Heart
Source: PLoS One. 2016 May 19;11(5):e0155611. doi: 10.1371/journal.pone.0155611 (PMC4873222; doi:10.1371/journal.pone.0155611)
Supplement: S2 Table — (PDF) [file pone.0155611.s005.pdf]

SUPPLEMENTARY DATA

Supplementary Table 2: List of primary antibodies used in the study.

**Primary antibody**	**Antibody reference, Dilution, Manufacturer**
Monoclonal mouse anti-mouse CD36	Antibody # ABM-5525, dilution 1:1000, Cascade (Winchester, MA),
Polyclonal goat-anti-human FATP1 (cross react with mouse protein)	Antibody # sc-31954, dilution 1:200, Santa Cruz Biotech.
Polyclonal rabbit anti-mouse Akt (cross react with mouse protein)	Antibody # 9272, 1:1000, Cell signaling-Millipore (Billerica, MA)
Polyclonal rabbit anti-mouse phospho-Akt (Ser473) (cross react with mouse protein)	Antibody # 9271, 1:1000, Cell signaling
Polyclonal rabbit anti-human IRS1 (cross reacts with mouse protein)	Antibody # 2382, 1:1000, Cell signaling
Polyclonal rabbit anti-mouse phospho-IRS1 (Tyr608), (cross react with mouse protein)	Antibody # 09-432, Upstate-Millipore
Polyclonal rabbit ant-human PKCα (cross reacts with mouse protein)	Antibody # 2056, Cell signaling, 1:1000
Polyclonal rabbit anti-human PKCδ (cross reacts with mouse protein)	Antibody # 2058, dilution, 1:1000, Cell signaling
Monoclonal mouse anti-mouse $p47^{phox}$	Antibody # sc-17845, dilution 1:100
Monoclonal rabbit anti-NOX2	Antibody # ab129068, dilution 1: 2500, Abcam (Cambridge, MA)
Monoclonal rabbit anti-Nox4	Antibody # ab133303, dilution 1:2500, Abcam
Monoclonal mouse anti-human $p67^{phox}$ cross react with mouse	Antibody # sc-374510, 1:200, Santa Cruz Biotech.
Polyclonal rabbit anti-human $p22^{phox}$ cross react with mouse	Antibody # sc-20781, dilution 1:200, Santa Cruz
Polyclonal rabbit anti human heart-FABP (cross react with mouse protein)	Antibody # ab102075, dilution 1:2000, Abcam
Polyclonal rabbit anti human PPARα (cross react with mouse)	Antibody # 3585-100, dilution 1:1000, BioVision (Mountview, CA)

Secondary antibodies were selected according to the host of the primary antibodies. The following antibodies were used: Polyclonal rabbit anti-mouse IgG, peroxidase conjugated (sc-358923, Santa Cruz Biotech.), donkey anti-goat IgG-peroxidase conjugated (sc-2033) and goat anti-rabbit IgG-peroxidase (A0545)
