# Supplementary material for: Cluster Differentiating 36 (CD36) Deficiency Attenuates Obesity-Associated Oxidative Stress in the Heart
Source: PLoS One. 2016 May 19;11(5):e0155611. doi: 10.1371/journal.pone.0155611 (PMC4873222; doi:10.1371/journal.pone.0155611)
Supplement: S3 Table — (PDF) [file pone.0155611.s006.pdf]

SUPPLEMENTARY DATA – TABLE 3

Figure 7 data and stats

Mean, SD, n

	WT	SD	n	Ob	SD	n	Ob_CD36ko	SD	n
**Chemi**									
Basal	229.600	60.47148	5	626.000	95.05524	5	329.600	75.94933	5
SOD	20.400	9.154234	5	26.800	15.18881	5	21.800	17.54138	5
L-Name	212.200	60.05581	5	592.000	98.4378	5	297.200	67.1878	5
Oxyp	214.000	59.31273	5	573.200	85.95173	5	292.600	74.62439	5
Rotenone	158.800	42.48176	5	407.200	81.56715	5	243.400	46.56501	5
Apocynin	59.200	22.18558	5	154.800	36.4123	5	172.200	38.16674	5
DPI	41.400	20.18167	5	124.800	34.53549	5	141.600	25.83215	5

Stats: Basal

Parameter	
Table Analyzed	Data 1
One-way analysis of variance	
P value	< 0.0001
P value summary	****
Are means signif. different? (P < 0.05)	Yes
Number of groups	3
F	34.53
R square	0.8520

ANOVA Table	SS	df	MS
Treatment (between columns)	424977	2	212488
Residual (within columns)	73842	12	6154
Total	498819	14	

Tukey's Multiple Comparison Test	Mean Diff.	q	Significant? P < 0.05?	Summary	95% CI of diff
WT vs Ob	-396.4	11.30	Yes	***	-528.8 to -264.0
WT vs Ob_CD36ko	-100.0	2.851	No	ns	-232.4 to 32.36
Ob vs Ob_CD36ko	296.4	8.449	Yes	***	164.0 to 428.8

Stats: SOD

Table Analyzed	Data 1
One-way analysis of variance	
P value	0.7658
P value summary	ns
Are means signif. different? (P < 0.05)	No
Number of groups	3
F	0.2729
R square	0.04350

ANOVA Table	SS	df	MS
Treatment (between columns)	113.2	2	56.60
Residual (within columns)	2489	12	207.4
Total	2602	14	

Tukey's Multiple Comparison Test	Mean Diff.	q	Significant? P < 0.05?	Summary	95% CI of diff
WT vs Ob	-6.400	0.9937	No	ns	-30.70 to 17.90
WT vs Ob_CD36ko	-1.400	0.2174	No	ns	-25.70 to 22.90
Ob vs Ob_CD36ko	5.000	0.7763	No	ns	-19.30 to 29.30

Stats: L-Name

Parameter	
Table Analyzed	Data 1
One-way analysis of variance	
P value	< 0.0001
P value summary	****
Are means signif. different? (P < 0.05)	Yes
Number of groups	3
F	33.46

R square	0.8479				

ANOVA Table	SS	df	MS
Treatment (between columns)	397300	2	198650
Residual (within columns)	71244	12	5937
Total	468544	14	

Tukey's Multiple Comparison Test	Mean Diff.	q	Significant? P < 0.05?	Summary	95% CI of diff
WT vs Ob	-379.8	11.02	Yes	***	-509.8 to -249.8
WT vs Ob_CD36ko	-85.00	2.467	No	ns	-215.0 to 45.01
Ob vs Ob_CD36ko	294.8	8.555	Yes	***	164.8 to 424.8

Stats: Oxypurinol

Parameter	
Table Analyzed	Data 1
One-way analysis of variance	
P value	< 0.0001
P value summary	****
Are means signif. different? (P < 0.05)	Yes
Number of groups	3
F	32.47
R square	0.8440

ANOVA Table	SS	df	MS
Treatment (between columns)	356565	2	178282
Residual (within columns)	65898	12	5491
Total	422463	14	

Tukey's Multiple Comparison Test	Mean Diff.	q	Significant? P < 0.05?	Summary	95% CI of diff
WT vs Ob	-359.2	10.84	Yes	***	-484.2 to -234.2
WT vs Ob_CD36ko	-78.60	2.372	No	ns	-203.6 to 46.44
Ob vs Ob_CD36ko	280.6	8.467	Yes	***	155.6 to 405.6

Stats: Rotenone

Parameter	
Table Analyzed	Data 1
One-way analysis of variance	
P value	< 0.0001
P value summary	****
Are means signif. different? (P < 0.05)	Yes
Number of groups	3
F	22.51
R square	0.7896

ANOVA Table	SS	df	MS
Treatment (between columns)	159484	2	79742
Residual (within columns)	42505	12	3542
Total	201988	14	

Tukey's Multiple Comparison Test	Mean Diff.	q	Significant? P < 0.05?	Summary	95% CI of diff
WT vs Ob	-248.4	9.333	Yes	***	-348.8 to -148.0
WT vs Ob_CD36ko	-84.60	3.179	No	ns	-185.0 to 15.82
Ob vs Ob_CD36ko	163.8	6.154	Yes	**	63.38 to 264.2

Stats: Apocynin

Parameter	
Table Analyzed	Data 1
One-way analysis of variance	
P value	0.0003
P value summary	***
Are means signif. different? (P < 0.05)	Yes
Number of groups	3
F	16.93
R square	0.7383

ANOVA Table	SS	df	MS
Treatment (between columns)	37019	2	18509
Residual (within columns)	13120	12	1093
Total	50139	14	

Tukey's Multiple Comparison Test	Mean Diff.	q	Significant? P < 0.05?	Summary	95% CI of diff
WT vs Ob	-95.60	6.465	Yes	**	-151.4 to -39.81
WT vs Ob_CD36ko	-113.0	7.642	Yes	***	-168.8 to -57.21
Ob vs Ob_CD36ko	-17.40	1.177	No	ns	-73.19 to 38.39

Stats: DPI

Parameter	
Table Analyzed	Data 1
One-way analysis of variance	
P value	0.0002
P value summary	***
Are means signif. different? (P < 0.05)	Yes
Number of groups	3
F	19.05
R square	0.7605

ANOVA Table	SS	df	MS
Treatment (between columns)	28796	2	14398
Residual (within columns)	9069	12	755.8
Total	37866	14	

Tukey's Multiple Comparison Test	Mean Diff.	q	Significant? P < 0.05?	Summary	95% CI of diff
WT vs Ob	-83.40	6.784	Yes	**	-129.8 to -37.01
WT vs Ob_CD36ko	-100.2	8.150	Yes	***	-146.6 to -53.81
Ob vs Ob_CD36ko	-16.80	1.366	No	ns	-63.19 to 29.59
