# Supplementary material for: Cluster Differentiating 36 (CD36) Deficiency Attenuates Obesity-Associated Oxidative Stress in the Heart
Source: PLoS One. 2016 May 19;11(5):e0155611. doi: 10.1371/journal.pone.0155611 (PMC4873222; doi:10.1371/journal.pone.0155611)
Supplement: S4 Table — (PDF) [file pone.0155611.s007.pdf]

SUPPLEMENTARY DATA – TABLE 4

Figure 1C: FDG uptake

Mean, SD, n

	WT			OB			OB-CDKO		
	Mean	SD	n	Mean	SD	n	Mean	SD	n
FDG Heart	61.500	12.81796	6	30.000	6.811755	6	58.000	10.11929	6
FDG Sk. Muscle	12.550	3.482959	6	4.800	1.834121	6	8.883333	1.729065	6
FDG Adipose T	7.033333	1.728198	6	2.866667	1.312504	6	5.456667	1.029984	6
FDG Liver	15.68333	2.231965	6	8.183333	2.142351	6	4.183333	1.195687	6

Stats Heart FDG uptake: 1 way ANOVA and Tukey's test

Parameter	
Table Analyzed	Data 1
One-way analysis of variance	
P value	0.0001
P value summary	***
Are means signif. different? (P < 0.05)	Yes
Number of groups	3
F	17.14
R square	0.6956

ANOVA Table	SS	df	MS
Treatment (between columns)	3577	2	1789
Residual (within columns)	1566	15	104.4
Total	5143	17	

Tukey's Multiple Comparison Test	Mean Diff.	q	Significant? P < 0.05?	Summary	95% CI of diff
WT vs OB	31.50	7.553	Yes	***	16.18 to 46.82
WT vs OB-CDKO	3.500	0.8392	No	ns	-11.82 to 18.82
OB vs OB-CDKO	-28.00	6.714	Yes	***	-43.32 to -12.68

Stats Sk. muscle FDG: 1 way ANOVA and Tukey's test

Parameter	
Table Analyzed	Data 1
One-way analysis of variance	
P value	0.0003
P value summary	***
Are means signif. different? (P < 0.05)	Yes
Number of groups	3
F	14.64
R square	0.6612

ANOVA Table	SS	df	MS
Treatment (between columns)	180.4	2	90.18
Residual (within columns)	92.42	15	6.162
Total	272.8	17	

Tukey's Multiple Comparison Test	Mean Diff.	q	Significant? P < 0.05?	Summary	95% CI of diff
WT vs OB	7.750	7.648	Yes	***	4.027 to 11.47
WT vs OB-CDKO	3.667	3.618	No	ns	-0.05647 to 7.390
OB vs OB-CDKO	-4.083	4.029	Yes	*	-7.806 to -0.3602

Stats Adipose FDG uptake: 1 way ANOVA and Tukey's test

Parameter	
Table Analyzed	Data 1
One-way analysis of variance	
P value	0.0004
P value summary	***
Are means signif. different? (P < 0.05)	Yes
Number of groups	3
F	13.81
R square	0.6480

ANOVA Table	SS	df	MS
Treatment (between columns)	53.11	2	26.56
Residual (within columns)	28.85	15	1.923
Total	81.96	17	

Tukey's Multiple Comparison Test	Mean Diff.	q	Significant? P < 0.05?	Summary	95% CI of diff
WT vs OB	4.167	7.359	Yes	***	2.087 to 6.247
WT vs OB-CDKO	1.577	2.785	No	ns	-0.5035 to 3.657
OB vs OB-CDKO	-2.590	4.574	Yes	*	-4.670 to -0.5098

Stats Liver FDG uptake: 1 way ANOVA and Tukey's test

Parameter					
Table Analyzed	Data 1				
One-way analysis of variance					
P value	< 0.0001				
P value summary	****				
Are means signif. different? (P < 0.05)	Yes				
Number of groups	3				
F	55.77				
R square	0.8815				
ANOVA Table	SS	df	MS		
Treatment (between columns)	409.0	2	204.5		
Residual (within columns)	55.00	15	3.667		
Total	464.0	17			
Tukey's Multiple Comparison Test	Mean Diff.	q	Significant? P < 0.05?	Summary	95% CI of diff
WT vs OB	7.500	9.594	Yes	***	4.628 to 10.37
WT vs OB-CDKO	11.50	14.71	Yes	***	8.628 to 14.37
OB vs OB-CDKO	4.000	5.117	Yes	**	1.128 to 6.872

Figure 3A: Heart lipid content

Mean, SD, n

	WT			Ob			Ob-CD36KO		
Heart TG (ug/mg prot)	Mean	SD	n	Mean	SD	n	Mean	SD	n
TG	2.754286	1.341962	7	8.733334	1.935631	6	4.458333	1.169349	6
FA	129.2857	47.70994	7	328.6833	87.9941	6	194.6667	72.61588	6
PL	16.61429	3.10238	7	15.18333	5.518847	6	14.16667	3.240782	6

Stats TG content: 1 way ANOVA and Turkey's test

Parameter					
Table Analyzed	Data 1				
One-way analysis of variance					
P value	< 0.0001				
P value summary	****				
Are means signif. different? (P < 0.05)	Yes				
Number of groups	3				
F	26.41				
R square	0.7675				
ANOVA Table	SS	df	MS		
Treatment (between columns)	120.1	2	60.04		
Residual (within columns)	36.38	16	2.273		
Total	156.4	18			
Tukey's Multiple Comparison Test	Mean Diff.	q	Significant? P < 0.05?	Summary	95% CI of diff
WT vs Ob	-5.979	10.08	Yes	***	-8.144 to -3.815
WT vs Ob-CD36KO	-1.704	2.873	No	ns	-3.869 to 0.4604
Ob vs Ob-CD36KO	4.275	6.945	Yes	***	2.029 to 6.521

Stats FA content: 1 way ANOVA and Turkey's test

Parameter					
Table Analyzed	Data 1				
One-way analysis of variance					
P value	0.0004				
P value summary	***				
Are means signif. different? (P < 0.05)	Yes				
Number of groups	3				
F	13.35				
R square	0.6252				
ANOVA Table	SS	df	MS		
Treatment (between columns)	131369	2	65684		
Residual (within columns)	78738	16	4921		
Total	210106	18			
Tukey's Multiple Comparison Test	Mean Diff.	q	Significant? P < 0.05?	Summary	95% CI of diff
WT vs Ob	-199.4	7.225	Yes	***	-300.1 to -98.70
WT vs Ob-CD36KO	-65.38	2.369	No	ns	-166.1 to 35.32
Ob vs Ob-CD36KO	134.0	4.680	Yes	*	29.51 to 238.5

Stats PL content: 1 way ANOVA and Turkey's test

Parameter	
Table Analyzed	Data 1
One-way analysis of variance	
P value	0.5601
P value summary	ns
Are means signif. different? (P < 0.05)	No
Number of groups	3
F	0.6011
R square	0.06989

ANOVA Table	SS	df	MS
Treatment (between columns)	19.73	2	9.864
Residual (within columns)	262.6	16	16.41
Total	282.3	18	

Tukey's Multiple Comparison Test	Mean Diff.	q	Significant? P < 0.05?	Summary	95% CI of diff
WT vs Ob	1.431	0.8979	No	ns	-4.384 to 7.246
WT vs Ob-CD36KO	2.448	1.536	No	ns	-3.367 to 8.263
Ob vs Ob-CD36KO	1.017	0.6148	No	ns	-5.018 to 7.051

Figure 3C: BMIPP incorporation

Mean, SD, n

	WT			OB			OB-CDKO		
	Mean	SD	n	Mean	SD	n	Mean	SD	n
Polar	12.84714	4.435075	7	15.58333	4.438205	6	13.73333	3.55509	6
FA	8.785714	4.040391	7	16.150	5.331697	6	9.683333	3.487358	6
DG	24.55714	12.70602	7	48.98333	14.81653	6	27.800	8.188285	6
TG	37.000	9.273619	7	78.66666	13.85159	6	43.66667	10.01332	6

Stats TG: 1 way ANOVA and Turkey’s test

Parameter	
Table Analyzed	Data 1
One-way analysis of variance	
P value	< 0.0001
P value summary	****
Are means signif. different? (P < 0.05)	Yes
Number of groups	3
F	25.32
R square	0.7599

ANOVA Table	SS	df	MS
Treatment (between columns)	6257	2	3129
Residual (within columns)	1977	16	123.5
Total	8234	18	

Tukey's Multiple Comparison Test	Mean Diff.	q	Significant? P < 0.05?	Summary	95% CI of diff
WT vs Ob	-41.67	9.529	Yes	***	-57.62 to -25.71
WT vs Ob-CD36KO	-6.667	1.525	No	ns	-22.62 to 9.289
Ob vs Ob-CD36KO	35.00	7.713	Yes	***	18.44 to 51.56

Stats DG: 1 way ANOVA and Turkey’s test

Parameter	
Table Analyzed	Data 1
One-way analysis of variance	
P value	0.0056
P value summary	**
Are means signif. different? (P < 0.05)	Yes
Number of groups	3
F	7.303
R square	0.4772

ANOVA Table	SS	df	MS
Treatment (between columns)	2192	2	1096
Residual (within columns)	2402	16	150.1
Total	4594	18	

Tukey's Multiple Comparison Test	Mean Diff.	q	Significant? P < 0.05?	Summary	95% CI of diff
WT vs Ob	-24.43	5.068	Yes	**	-42.01 to -6.839
WT vs Ob-CD36KO	-3.243	0.6728	No	ns	-20.83 to 14.34
Ob vs Ob-CD36KO	21.18	4.235	Yes	*	2.932 to 39.43

Stats FA: 1 way ANOVA and Turkey’s test

Parameter	
Table Analyzed	Data 1
One-way analysis of variance	
P value	0.0167
P value summary	*
Are means signif. different? (P < 0.05)	Yes
Number of groups	3
F	5.341
R square	0.4004

ANOVA Table	SS	df	MS
Treatment (between columns)	200.9	2	100.4
Residual (within columns)	300.9	16	18.81
Total	501.8	18	

Tukey's Multiple Comparison Test	Mean Diff.	q	Significant? P < 0.05?	Summary	95% CI of diff
WT vs Ob	-7.364	4.317	Yes	*	-13.59 to -1.139
WT vs Ob-CD36KO	-0.8976	0.5262	No	ns	-7.123 to 5.328
Ob vs Ob-CD36KO	6.467	3.653	Yes	*	0.006503 to 12.93

Figure 3B: BMIPP uptake

Mean, SD, n

BMIPP % /dose/g wt	WT	SD	n	Ob	SD	n	Ob-CD36KO	SD	n
Heart	1.095714	0.2692494	7	2.225	0.3158323	6	1.386667	0.217501	6
Sk. musc	0.2328571	0.1264535	7	0.430	0.0997998	6	0.2583333	0.1018659	6
Adipose T	0.4285714	0.1524717	7	0.6451667	0.1103384	6	0.3566667	0.1490861	6
Liver	0.5728571	0.1059425	7	0.4216667	0.1200694	6	0.770	0.1694698	6

Stats: 1 way ANOVA and Tukey's test

Heart BMIPP uptake

Parameter	
Table Analyzed	Data 1
One-way analysis of variance	
P value	< 0.0001
P value summary	****
Are means signif. different? (P < 0.05)	Yes
Number of groups	3
F	29.65
R square	0.7875

ANOVA Table	SS	df	MS
Treatment (between columns)	4.338	2	2.169
Residual (within columns)	1.170	16	0.07314
Total	5.508	18	

Tukey's Multiple Comparison Test	Mean Diff.	q	Significant? P < 0.05?	Summary	95% CI of diff
WT vs Ob	-1.129	10.61	Yes	***	-1.518 to -0.7411
WT vs Ob-CD36KO	-0.2910	2.735	No	ns	-0.6792 to 0.09727
Ob vs Ob-CD36KO	0.8383	7.593	Yes	***	0.4355 to 1.241

Sk. Muscle

Parameter	
Table Analyzed	Data 1
One-way analysis of variance	
P value	0.0128
P value summary	*
Are means signif. different? (P < 0.05)	Yes
Number of groups	3
F	5.796
R square	0.4201

ANOVA Table	SS	df	MS
Treatment (between columns)	0.1432	2	0.07159
Residual (within columns)	0.1976	16	0.01235
Total	0.3408	18	

Tukey's Multiple Comparison Test	Mean Diff.	q	Significant? P < 0.05?	Summary	95% CI of diff
WT vs Ob	-0.1971	4.509	Yes	*	-0.3567 to -0.03760
WT vs Ob-CD36KO	-0.02548	0.5827	No	ns	-0.1850 to 0.1341
Ob vs Ob-CD36KO	0.1717	3.784	Yes	*	0.006105 to 0.3372

Adipose tissue

Parameter	
Table Analyzed	Data 1
One-way analysis of variance	
P value	0.0065
P value summary	**
Are means signif. different? (P < 0.05)	Yes
Number of groups	3
F	7.007
R square	0.4669

ANOVA Table	SS	df	MS
Treatment (between columns)	0.2728	2	0.1364
Residual (within columns)	0.3115	16	0.01947
Total	0.5843	18	

Tukey's Multiple Comparison Test	Mean Diff.	q	Significant? P < 0.05?	Summary	95% CI of diff
WT vs Ob	-0.2166	3.946	Yes	*	-0.4169 to -0.01630
WT vs Ob-CD36KO	0.07190	1.310	No	ns	-0.1284 to 0.2722
Ob vs Ob-CD36KO	0.2885	5.065	Yes	**	0.08064 to 0.4964

Liver BMIPP uptake

Parameter	
Table Analyzed	Data 1
One-way analysis of variance	
P value	< 0.0001
P value summary	****
Are means signif. different? (P < 0.05)	Yes
Number of groups	3

F	26.87					
R square	0.7818					

ANOVA Table	SS	df	MS
Treatment (between columns)	12304	2	6152
Residual (within columns)	3435	15	229.0
Total	15739	17	

Tukey's Multiple Comparison Test	Mean Diff.	q	Significant? P < 0.05?	Summary	95% CI of diff
WT vs Ob	-59.45	9.623	Yes	***	-82.15 to -36.75
WT vs Ob-CD36KO	-9.100	1.473	No	ns	-31.80 to 13.60
Ob vs Ob-CD36KO	50.35	8.150	Yes	***	27.65 to 73.05

Figure 4A: FA uptake in vitro

Mean, SD, n

FA uptake	WT			Ob			Ob-CD36KO		
	Mean	SD	n	Mean	SD	n	Mean	SD	n
	27.700	8.757854	6	88.81667	15.70776	6	38.46667	12.39107	6

Stats FA uptake in vitro: 1 way ANOVA and Tukey's test

Parameter	
Table Analyzed	Data 1
One-way analysis of variance	
P value	< 0.0001
P value summary	****
Are means signif. different? (P < 0.05)	Yes
Number of groups	3
F	40.17
R square	0.8427

ANOVA Table	SS	df	MS
Treatment (between columns)	12773	2	6386
Residual (within columns)	2385	15	159.0
Total	15157	17	

Tukey's Multiple Comparison Test	Mean Diff.	q	Significant? P < 0.05?	Summary	95% CI of diff
WT vs Ob	-61.12	11.87	Yes	***	-80.03 to -42.20
WT vs Ob-CD36KO	-10.77	2.092	No	ns	-29.68 to 8.146
Ob vs Ob-CD36KO	50.35	9.781	Yes	***	31.44 to 69.26

Figure 4B: FA oxidation in vitro

Mean, SD, n

FA oxidation	**WT**	**SD**	**n**	**Ob**	**SD**	**n**	**Ob-CD36KO**	**SD**	**n**
	8.950	3.195466	6	25.41667	5.531516	6	12.83333	3.920544	6

Stats FA oxidation: 1 way ANOVA and Tukey's test

Parameter	
Table Analyzed	Data 1
One-way analysis of variance	
P value	< 0.0001
P value summary	****
Are means signif. different? (P < 0.05)	Yes
Number of groups	3
F	23.74
R square	0.7599

ANOVA Table	SS	df	MS
Treatment (between columns)	889.1	2	444.6
Residual (within columns)	280.9	15	18.73
Total	1170	17	

Tukey's Multiple Comparison Test	Mean Diff.	q	Significant? P < 0.05?	Summary	95% CI of diff
WT vs Ob	-16.47	9.321	Yes	***	-22.96 to -9.976
WT vs Ob-CD36KO	-3.883	2.198	No	ns	-10.37 to 2.607
Ob vs Ob-CD36KO	12.58	7.123	Yes	***	6.093 to 19.07

Figure 4C: Glucose uptake

Mean, SD, n

Glucose uptake	WT	SD	n	Ob	SD	n	Ob-CD36KO	SD	n
0 ins	74.33334	44.96962	6	31.16667	16.59418	6	55.000	21.86321	6
10 nM ins	620.8333	134.4655	6	195.6667	81.62025	6	469.500	116.4899	6

Stats: Glucose uptake without Insulin (0 Ins)

Parameter					
Table Analyzed	Data 1				
One-way analysis of variance					
P value	0.0784				
P value summary	ns				
Are means signif. different? (P < 0.05)	No				
Number of groups	3				
F	3.032				
R square	0.2879				
ANOVA Table	SS	df	MS		
Treatment (between columns)	5610	2	2805		
Residual (within columns)	13878	15	925.2		
Total	19489	17			
Tukey's Multiple Comparison Test	Mean Diff.	q	Significant? P < 0.05?	Summary	95% CI of diff
WT vs Ob	43.17	3.476	No	ns	-2.456 to 88.79
WT vs Ob-CD36KO	19.33	1.557	No	ns	-26.29 to 64.96
Ob vs Ob-CD36KO	-23.83	1.919	No	ns	-69.46 to 21.79

Stats: Glucose uptake with Insulin

Parameter					
Table Analyzed	Data 1				
One-way analysis of variance					
P value	< 0.0001				
P value summary	****				
Are means signif. different? (P < 0.05)	Yes				
Number of groups	3				
F	25.67				
R square	0.7739				
ANOVA Table	SS	df	MS		
Treatment (between columns)	599425	2	299712		
Residual (within columns)	175137	15	11676		
Total	774562	17			
Tukey's Multiple Comparison Test	Mean Diff.	q	Significant? P < 0.05?	Summary	95% CI of diff
WT vs Ob	433.5	9.827	Yes	***	271.4 to 595.6
WT vs Ob-CD36KO	122.3	2.773	No	ns	-39.74 to 284.4
Ob vs Ob-CD36KO	-311.2	7.054	Yes	***	-473.2 to -149.1

Figure 4D: Glucose oxidation in vitro

Mean, SD, n

	WT	SD	n	Ob	SD	n	Ob-CD36KO	SD	n
Glucose oxidation	10.985	2.13664	6	3.568333	1.277222	6	8.278334	2.23464	6

Stats Glucose oxidation: 1 way ANOVA and Tukey's test

Parameter					
Table Analyzed	Data 1				
One-way analysis of variance					
P value	< 0.0001				
P value summary	****				
Are means signif. different? (P < 0.05)	Yes				
Number of groups	3				
F	22.66				
R square	0.7513				
ANOVA Table	SS	df	MS		
Treatment (between columns)	169.0	2	84.52		
Residual (within columns)	55.95	15	3.730		
Total	225.0	17			
Tukey's Multiple Comparison Test	Mean Diff.	q	Significant? P < 0.05?	Summary	95% CI of diff
WT vs Ob	7.417	9.406	Yes	***	4.520 to 10.31
WT vs Ob-CD36KO	2.707	3.433	No	ns	-0.1901 to 5.603
Ob vs Ob-CD36KO	-4.710	5.974	Yes	**	-7.607 to -1.813

Figure 5B: Heart gene expression:

FATP1

Mean, SD, n

mRNA FATP1	WT	SD	n	Ob	SD	n	Ob-CD36KO	SD	n
	1.000	0.2408316	6	2.34405	0.4411497	6	1.256397	0.2866619	6

One-way ANOVA test and Tukey’s test

Parameter	
Table Analyzed	Data 1
One-way analysis of variance	
P value	< 0.0001
P value summary	****
Are means signif. different? (P < 0.05)	Yes
Number of groups	3
F	27.37
R square	0.7849

ANOVA Table	SS	df	MS
Treatment (between columns)	6.108	2	3.054
Residual (within columns)	1.674	15	0.1116
Total	7.782	17	

Tukey's Multiple Comparison Test	Mean Diff.	q	Significant? P < 0.05?	Summary	95% CI of diff
WT vs Ob	-1.344	9.853	Yes	***	-1.845 to -0.8427
WT vs Ob-CD36KO	-0.2561	1.878	No	ns	-0.7571 to 0.2450
Ob vs Ob-CD36KO	1.088	7.975	Yes	***	0.5866 to 1.589

H-FABP

Mean, SD, n

mRNA FABP1	WT	SD	n	Ob	SD	n	Ob-CD36KO	SD	n
	1.000333	0.1554615	6	2.345667	1.050116	6	1.36885	0.1612766	6

One-way ANOVA test and Tukey’s test

Parameter	
Table Analyzed	Data 1
One-way analysis of variance	
P value	0.0054
P value summary	**
Are means signif. different? (P < 0.05)	Yes
Number of groups	3
F	7.546
R square	0.5015

ANOVA Table	SS	df	MS
Treatment (between columns)	5.800	2	2.900
Residual (within columns)	5.765	15	0.3843
Total	11.56	17	

Tukey's Multiple Comparison Test	Mean Diff.	q	Significant? P < 0.05?	Summary	95% CI of diff
WT vs Ob	-1.345	5.316	Yes	**	-2.275 to -0.4155
WT vs Ob-CD36KO	-0.3685	1.456	No	ns	-1.298 to 0.5613
Ob vs Ob-CD36KO	0.9768	3.860	Yes	*	0.04699 to 1.907

PPARα

Mean, SD, n

mRNA PPARa	WT	SD	n	Ob	SD	n	Ob-CD36KO	SD	n
	1.00000	0.3649636	6	2.531183	0.6323144	6	1.65905	0.7186134	6

One-way ANOVA test and Tukey’s test

Parameter	
Table Analyzed	Data 1
One-way analysis of variance	
P value	0.0017
P value summary	**
Are means signif. different? (P < 0.05)	Yes
Number of groups	3
F	10.11
R square	0.5742

ANOVA Table	SS	df	MS
Treatment (between columns)	7.075	2	3.538
Residual (within columns)	5.247	15	0.3498

Total	12.32	17			
Tukey's Multiple Comparison Test	Mean Diff.	q	Significant? P < 0.05?	Summary	95% CI of diff
WT vs Ob	-1.531	6.340	Yes	**	-2.418 to -0.6436
WT vs Ob-CD36KO	-0.6586	2.728	No	ns	-1.546 to 0.2285
Ob vs Ob-CD36KO	0.8721	3.612	No	ns	-0.01498 to 1.759

Figure 6:

Figure 6A: Isoprostanes: Mean, SD, n, One-way ANOVA test and Tukey's test

	WT	SD	n	Ob	SD	n	Ob-CD36KO	SD	n
Isoprotanes (ng/g wt)	2.621667	0.9727985	6	10.77333	1.565243	6	3.943333	1.710598	6

Stats, Isoprostanes

Parameter					
Table Analyzed	Data 1				
One-way analysis of variance					
P value	< 0.0001				
P value summary	****				
Are means signif. different? (P < 0.05)	Yes				
Number of groups	3				
F	54.49				
R square	0.8790				
ANOVA Table	SS	df	MS		
Treatment (between columns)	229.7	2	114.8		
Residual (within columns)	31.61	15	2.107		
Total	261.3	17			
Tukey's Multiple Comparison Test	Mean Diff.	q	Significant? P < 0.05?	Summary	95% CI of diff
WT vs Ob	-8.152	13.75	Yes	***	-10.33 to -5.974
WT vs Ob-CD36KO	-1.322	2.230	No	ns	-3.499 to 0.8558
Ob vs Ob-CD36KO	6.830	11.52	Yes	***	4.653 to 9.007

Figure 6B: LPO Mean, SD, SE, One-way ANOVA test and Tukey's test

	WT	SD	n	Ob	SD	n	Ob-CD36KO	SD	n
LPO (nmol/g wt)	2.423333	0.9916095	6	6.841667	1.2780303	6	3.188333	1.3260168	6

Stats LPO

Parameter					
Table Analyzed	Data 1				
One-way analysis of variance					
P value	< 0.0001				
P value summary	****				
Are means signif. different? (P < 0.05)	Yes				
Number of groups	3				
F	22.92				
R square	0.7534				
ANOVA Table	SS	df	MS		
Treatment (between columns)	66.95	2	33.47		
Residual (within columns)	21.91	15	1.461		
Total	88.86	17			
Tukey's Multiple Comparison Test	Mean Diff.	q	Significant? P < 0.05?	Summary	95% CI of diff
WT vs Ob	-4.418	8.955	Yes	***	-6.231 to -2.606
WT vs Ob-CD36KO	-0.7617	1.544	No	ns	-2.574 to 1.051
Ob vs Ob-CD36KO	3.657	7.411	Yes	***	1.844 to 5.469

Figure 6C: Hydrogen peroxides Mean, SD, n, One-way ANOVA test and Tukey's test

	WT	SD	n	Ob	SD	n	Ob-CD36KO	SD	n
H Perox Amplex (nmol/g wt)	100.3333	33.35066	6	368.500	59.4668	6	171.1667	57.08736	6

Stats Hydrogen peroxides

Parameter					
Table Analyzed	Data 1				
One-way analysis of variance					
P value	< 0.0001				
P value summary	****				
Are means signif. different? (P < 0.05)	Yes				
Number of groups	3				
F	43.96				
R square	0.8543				
ANOVA Table	SS	df	MS		
Treatment (between columns)	231742	2	115871		
Residual (within columns)	39538	15	2636		
Total	271280	17			
Tukey's Multiple Comparison Test	Mean Diff.	q	Significant? P < 0.05?	Summary	95% CI of diff
WT vs Ob	-268.2	12.79	Yes	***	-345.2 to -191.2
WT vs Ob-CD36KO	-70.83	3.380	No	ns	-147.8 to 6.172

Ob vs Ob-CD36KO	197.3	9.415	Yes	***	120.3 to 274.3

Figure 7 data and stats

Figure 7A: Mean, SD, n, One-way ANOVA test and Tukey’s test

Chemi	WT	SD	n	Ob	SD	n	Ob_CD36ko	SD	n
Basal	229.600	60.47148	5	626.000	95.05524	5	329.600	75.94933	5
SOD	20.400	9.154234	5	26.800	15.18881	5	21.800	17.54138	5
L-Name	212.200	60.05581	5	592.000	98.4378	5	297.200	67.1878	5
Oxyp	214.000	59.31273	5	573.200	85.95173	5	292.600	74.62439	5
Rotenone	158.800	42.48176	5	407.200	81.56715	5	243.400	46.56501	5
Apocynin	59.200	22.18558	5	154.800	36.4123	5	172.200	38.16674	5
DPI	41.400	20.18167	5	124.800	34.53549	5	141.600	25.83215	5

Stats: Basal

Parameter	
Table Analyzed	Data 1
One-way analysis of variance	
P value	< 0.0001
P value summary	****
Are means signif. different? (P < 0.05)	Yes
Number of groups	3
F	34.53
R square	0.8520

ANOVA Table	SS	df	MS
Treatment (between columns)	424977	2	212488
Residual (within columns)	73842	12	6154
Total	498819	14	

Tukey's Multiple Comparison Test	Mean Diff.	q	Significant? P < 0.05?	Summary	95% CI of diff
WT vs Ob	-396.4	11.30	Yes	***	-528.8 to -264.0
WT vs Ob_CD36ko	-100.0	2.851	No	ns	-232.4 to 32.36
Ob vs Ob_CD36ko	296.4	8.449	Yes	***	164.0 to 428.8

Stats: SOD

Table Analyzed	Data 1
One-way analysis of variance	
P value	0.7658
P value summary	ns
Are means signif. different? (P < 0.05)	No
Number of groups	3
F	0.2729
R square	0.04350

ANOVA Table	SS	df	MS
Treatment (between columns)	113.2	2	56.60
Residual (within columns)	2489	12	207.4
Total	2602	14	

Tukey's Multiple Comparison Test	Mean Diff.	q	Significant? P < 0.05?	Summary	95% CI of diff
WT vs Ob	-6.400	0.9937	No	ns	-30.70 to 17.90
WT vs Ob_CD36ko	-1.400	0.2174	No	ns	-25.70 to 22.90
Ob vs Ob_CD36ko	5.000	0.7763	No	ns	-19.30 to 29.30

Stats: L-Name

Parameter	
Table Analyzed	Data 1
One-way analysis of variance	
P value	< 0.0001
P value summary	****
Are means signif. different? (P < 0.05)	Yes
Number of groups	3
F	33.46
R square	0.8479

ANOVA Table	SS	df	MS
Treatment (between columns)	397300	2	198650
Residual (within columns)	71244	12	5937
Total	468544	14	

Tukey's Multiple Comparison Test	Mean Diff.	q	Significant? P < 0.05?	Summary	95% CI of diff
WT vs Ob	-379.8	11.02	Yes	***	-509.8 to -249.8
WT vs Ob_CD36ko	-85.00	2.467	No	ns	-215.0 to 45.01
Ob vs Ob_CD36ko	294.8	8.555	Yes	***	164.8 to 424.8

Stats: Oxypurinol

Parameter	
Table Analyzed	Data 1
One-way analysis of variance	
P value	< 0.0001
P value summary	****

Are means signif. different? (P < 0.05)	Yes				
Number of groups	3				
F	32.47				
R square	0.8440				
ANOVA Table	SS	df	MS		
Treatment (between columns)	356565	2	178282		
Residual (within columns)	65898	12	5491		
Total	422463	14			
Tukey's Multiple Comparison Test	Mean Diff.	q	Significant? P < 0.05?	Summary	95% CI of diff
WT vs Ob	-359.2	10.84	Yes	***	-484.2 to -234.2
WT vs Ob_CD36ko	-78.60	2.372	No	ns	-203.6 to 46.44
Ob vs Ob_CD36ko	280.6	8.467	Yes	***	155.6 to 405.6

Stats: Rotenone

Parameter					
Table Analyzed	Data 1				
One-way analysis of variance					
P value	< 0.0001				
P value summary	****				
Are means signif. different? (P < 0.05)	Yes				
Number of groups	3				
F	22.51				
R square	0.7896				
ANOVA Table	SS	df	MS		
Treatment (between columns)	159484	2	79742		
Residual (within columns)	42505	12	3542		
Total	201988	14			
Tukey's Multiple Comparison Test	Mean Diff.	q	Significant? P < 0.05?	Summary	95% CI of diff
WT vs Ob	-248.4	9.333	Yes	***	-348.8 to -148.0
WT vs Ob_CD36ko	-84.60	3.179	No	ns	-185.0 to 15.82
Ob vs Ob_CD36ko	163.8	6.154	Yes	**	63.38 to 264.2

Stats: Apocynin

Parameter					
Table Analyzed	Data 1				
One-way analysis of variance					
P value	0.0003				
P value summary	***				
Are means signif. different? (P < 0.05)	Yes				
Number of groups	3				
F	16.93				
R square	0.7383				
ANOVA Table	SS	df	MS		
Treatment (between columns)	37019	2	18509		
Residual (within columns)	13120	12	1093		
Total	50139	14			
Tukey's Multiple Comparison Test	Mean Diff.	q	Significant? P < 0.05?	Summary	95% CI of diff
WT vs Ob	-95.60	6.465	Yes	**	-151.4 to -39.81
WT vs Ob_CD36ko	-113.0	7.642	Yes	***	-168.8 to -57.21
Ob vs Ob_CD36ko	-17.40	1.177	No	ns	-73.19 to 38.39

Stats: DPI

Parameter					
Table Analyzed	Data 1				
One-way analysis of variance					
P value	0.0002				
P value summary	***				
Are means signif. different? (P < 0.05)	Yes				
Number of groups	3				
F	19.05				
R square	0.7605				
ANOVA Table	SS	df	MS		
Treatment (between columns)	28796	2	14398		
Residual (within columns)	9069	12	755.8		
Total	37866	14			
Tukey's Multiple Comparison Test	Mean Diff.	q	Significant? P < 0.05?	Summary	95% CI of diff
WT vs Ob	-83.40	6.784	Yes	**	-129.8 to -37.01
WT vs Ob_CD36ko	-100.2	8.150	Yes	***	-146.6 to -53.81
Ob vs Ob_CD36ko	-16.80	1.366	No	ns	-63.19 to 29.59

Figure 9A:

WT +/- FA +/- VAS2870

Mean, SD, n

	WT (no Palmitate)	SD	n	WT + Palmitate	SD	n	WT+Palmitate + VAS2870		
WT Fluorescence	0.07242305	0.03392887	4	0.3606364	0.04926436	4	0.1730757	0.07086562	4

Stats WT +/- FA +/- VAS2870

Parameter			
Table Analyzed	Data 1		
One-way analysis of variance			
P value	0.0001		
P value summary	***		
Are means signif. different? (P < 0.05)	Yes		
Number of groups	3		
F	29.85		
R square	0.8690		
ANOVA Table	SS	df	MS
Treatment (between columns)	0.1712	2	0.08558
Residual (within columns)	0.02580	9	0.002867
Total	0.1970	11	

Tukey's Multiple Comparison Test	Mean Diff.	q	Significant? P < 0.05?	Summary	95% CI of diff
WT (no Palmitate) vs WT + Palmitate	-0.2882	10.77	Yes	***	-0.3939 to -0.1825
WT (no Palmitate) vs WT+Palmitate + VAS2870	-0.1007	3.760	No	ns	-0.2064 to 0.005065
WT + Palmitate vs WT+Palmitate + VAS2870	0.1876	7.006	Yes	**	0.08184 to 0.2933

Ob +/- FA +/- VAS2870

Mean, SD, SE

Fluorescence	Ob (no Palmitate)	SD	n	Ob + Palmitate	SD	n	Ob + Palmitate + VAS2870	SD	n
Ob	0.2797813	0.155815	4	1.49674	0.2630103	4	0.5446244	0.2504396	4

Stats Ob <u>+</u> FA <u>+</u> VAS2870

Parameter			
Table Analyzed	Data 1		
One-way analysis of variance			
P value	< 0.0001		
P value summary	****		
Are means signif. different? (P < 0.05)	Yes		
Number of groups	3		
F	31.47		
R square	0.8749		
ANOVA Table	SS	df	MS
Treatment (between columns)	3.277	2	1.638
Residual (within columns)	0.4685	9	0.05206
Total	3.745	11	

Tukey's Multiple Comparison Test	Mean Diff.	q	Significant? P < 0.05?	Summary	95% CI of diff
Ob (no Palmitate) vs Ob + Palmitate	-1.217	10.67	Yes	***	-1.667 to -0.7665
Ob (no Palmitate) vs Ob + Palmitate + VAS2870	-0.2648	2.322	No	ns	-0.7153 to 0.1857
Ob + Palmitate vs Ob + Palmitate + VAS2870	0.9521	8.346	Yes	***	0.5016 to 1.403

Ob CD36ko +/- FA +/- VAS2870

Fluorescence	Ob-CD36ko (no Palmitate)			Ob-CD36ko + Palmitate			Ob-CD36k + Palmitate + VAS2870		
ob-CD36ko	0.1592283	0.06263548	4	0.5162992	0.106616	4	0.3639495	0.09415869	4

Stats

Parameter			
Table Analyzed	Data 1		
One-way analysis of variance			
P value	0.0011		
P value summary	**		
Are means signif. different? (P < 0.05)	Yes		
Number of groups	3		
F	15.95		
R square	0.7799		
ANOVA Table	SS	df	MS
Treatment (between columns)	0.2568	2	0.1284
Residual (within columns)	0.07247	9	0.008052
Total	0.3293	11	

Tukey's Multiple Comparison Test	Mean Diff.	q	Significant? P < 0.05?	Summary	95% CI of diff
Ob-CD36ko (no Palmitate) vs Ob-CD36ko + Palmitate	-0.3571	7.959	Yes	***	-0.5342 to -0.1799
Ob-CD36ko (no Palmitate) vs Ob-CD36k + Palmitate + VAS2870	-0.2047	4.563	Yes	*	-0.3819 to -0.02754
Ob-CD36ko + Palmitate vs Ob-CD36k + Palmitate + VAS2870	0.1523	3.396	No	ns	-0.02483 to 0.3295
